# Humans incorporate trial-to-trial working memory uncertainty into rewarded decisions

**DOI:** 10.1073/pnas.1918143117

**Published:** 2020-03-30

**Authors:** Maija Honig, Wei Ji Ma, Daryl Fougnie

**Affiliations:** ^a^Department of Psychology, New York University Center for Neural Science, New York, NY 10003;; ^b^Department of Psychology, New York University Abu Dhabi, Abu Dhabi, United Arab Emirates

**Keywords:** visual working memory, uncertainty, metamemory, reward, priors

## Abstract

Working memory (WM) plays an important role in action planning and decision making; however, both the informational content of memory and how that information is used in decisions remain poorly understood. To investigate this, we used a color WM task in which subjects viewed colored stimuli and reported both an estimate of a stimulus color and a measure of memory uncertainty, obtained through a rewarded decision. Reported memory uncertainty is correlated with memory error, showing that people incorporate their trial-to-trial memory quality into rewarded decisions. Moreover, memory uncertainty can be combined with other sources of information; after inducing expectations (prior beliefs) about stimuli probabilities, we found that estimates became shifted toward expected colors, with the shift increasing with reported uncertainty. The data are best fit by models in which people incorporate their trial-to-trial memory uncertainty with potential rewards and prior beliefs. Our results suggest that WM represents uncertainty information, and that this can be combined with prior beliefs. This highlights the potential complexity of WM representations and shows that rewarded decision can be a powerful tool for examining WM and informing and constraining theoretical, computational, and neurobiological models of memory.

## Significance

Information stored in working memory (WM) is incorporated into many daily decisions and actions, and many complex decisions involve WM; however, there has been little work on investigating what WM information is used in memory decisions. Here we try to draw connections between WM and decision making by manipulating prior beliefs in a standard WM task with rewards. We use this paradigm to show that WM contains a representation of the trial-by-trial uncertainty of visual stimuli. This uncertainty is incorporated into rewarded decisions along with other information, such as expectations about the environment. By studying WM in parallel with decision making, we can gain new insight into how these systems work together.

Working memory (WM), the storage and manipulation of information on a short timescale, is essential for many cognitive processes. For instance, individual differences in WM predict both intelligence and academic success (1–3). To understand WM, research has largely focused on examining the capacity and limitations of WM (4, 5), epitomized by such memory paradigms as delayed estimation (6–8), in which participants report a guess of a stimulus feature after a delay. In real life, however, WM information is used not only to make estimates of stimuli features, but also to make decisions and take actions. For example, when deciding when to cross the street, a person must remember from a glance the position and velocity of cars. Since a mistake in this decision is costly, and memories are noisy, WM information ought to be combined with information about potential rewards (getting to one’s destination sooner) and costs (getting hit by a car) of the decision. In doing so, it is useful to know how reliable or, conversely, how uncertain one’s memory is. If one is uncertain about the speed of an oncoming car, it would be wise to wait a little longer to avoid a high-cost collision.

Uncertainty in WM is rarely studied, however. There is evidence that individuals know something about the quality of their WM representations (9–11); for example, people can report which items from a set of stimuli they remember better (9). Beyond this, people know their uncertainty on a trial-to-trial level; when people make explicit reports of confidence in memory decisions, the amount of response error correlates with the reported confidence on each trial (10, 11). This could be explained by memory confidence being a function of internal fluctuations in underlying memory quality (12).

Confidence ratings are not necessarily a reflection of memory uncertainty (13, 14), however, and may be produced through a different mechanism from those used to make decisions under uncertainty (15, 16). Thus, to observe memory uncertainty, experimenters might not want to rely on explicit reports of uncertainty alone, but rather use paradigms in which people are incentivized to make decisions that implicitly incorporate memory uncertainty.

Integrating such WM uncertainty information into decisions would be highly adaptive; for example, when crossing the street at rush hour, when cars are likely to appear quickly, an observer ought to have a higher standard of certainty that a car is far away before crossing. Perceptual research has shown that people can improve their perceptual decisions by incorporating their uncertainty with other sensory information (17), rewards (18–20), or prior beliefs (21). However, although there may be a close connection between perceptual and WM representations (22–24), much less is known about the role of WM uncertainty in decisions. People combine WM reliability and peripheral sensory information to plan reaching movements (25) and perform nearly optimally in change detection tasks when items vary in reliability (26); however, in both perception and memory, these studies typically manipulate uncertainty by varying the encoding precision of stimuli (and thus the resulting memory uncertainty). This is done by manipulating the reliability of stimuli by varying such properties as contrast, density, and visual eccentricity. Nonetheless, participants can use cues about such attributes as a proxy for memory uncertainty without implicitly representing memory uncertainty (27, 28). In the present study, we aimed to show that even in the absence of explicit uncertainty manipulations, WM represents a trial-to-trial measure of uncertainty that is used in subsequent decisions.

## Results

### Experiment.

**Fig. 1.**
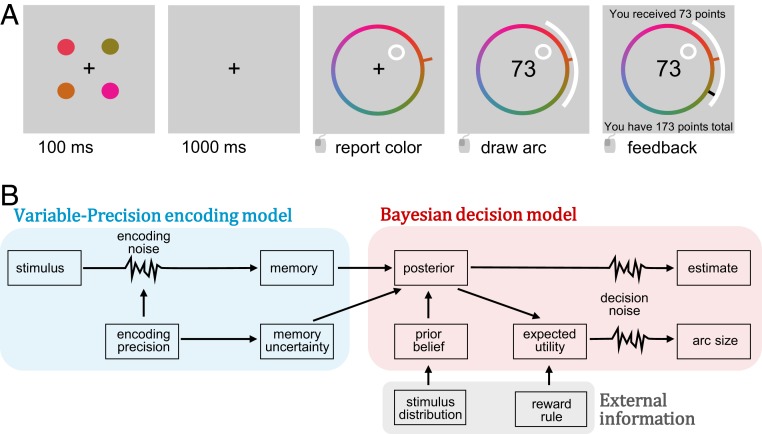
Experimental design. (*A*) Trial procedure. Participants viewed four colored stimuli. After a delay, they estimated the color of a probed stimulus on a color wheel and then drew a symmetric arc around their estimate. If the stimulus value fell outside of the arc, the participant received 0 points (“miss”); if it fell within (“hit”), the number of points received was a decreasing linear function of arc size. (*B*) Diagram of model structure.

To explore how WM information is taken into account in decisions, we modified a common WM paradigm, delayed estimation (6–8), to include a rewarded decision designed to reflect memory uncertainty. The experiment consisted of a sequence of memory trials in which participants viewed four colored circles and after a delay reported an estimate of the color of a certain probed circle. After responding, they were prompted to draw a symmetric “arc” around their estimate (Fig. 1*A*). We refer to this as the “arc size,” measured as half of the symmetric reported arc. If the true stimulus color was within the bounds of the arc (“hit”), the participant received points, which decreased linearly (100 to 0; “hit” points = 1 arc size/180; “miss” points = 0) as a function of arc size; otherwise, they received zero points (“miss”). This rule creates a trade-off between the probability of receiving a reward (as the arc grows, the stimulus is more likely to be inside it), and the magnitude of reward (as the arc grows, points from a hit decrease). Participants received monetary and time rewards based on points obtained, incentivizing them to use memory uncertainty when reporting arcs (*Methods*).

Furthermore, to test whether WM uncertainty could be integrated with other knowledge, we introduced expectations about stimuli colors. In two of the four experimental sessions, stimuli colors were drawn from a uniform stimulus distribution, while in the other two, colors were drawn from a von Mises (circular normal) distribution. Participants were taught the distributions through extensive training (*SI Appendix*, section S1*A*). In this experiment, combining WM uncertainty with the provided reward rule and the stimulus distribution would yield a greater reward.

### Uniform Stimulus Distribution.

**Fig. 2.**
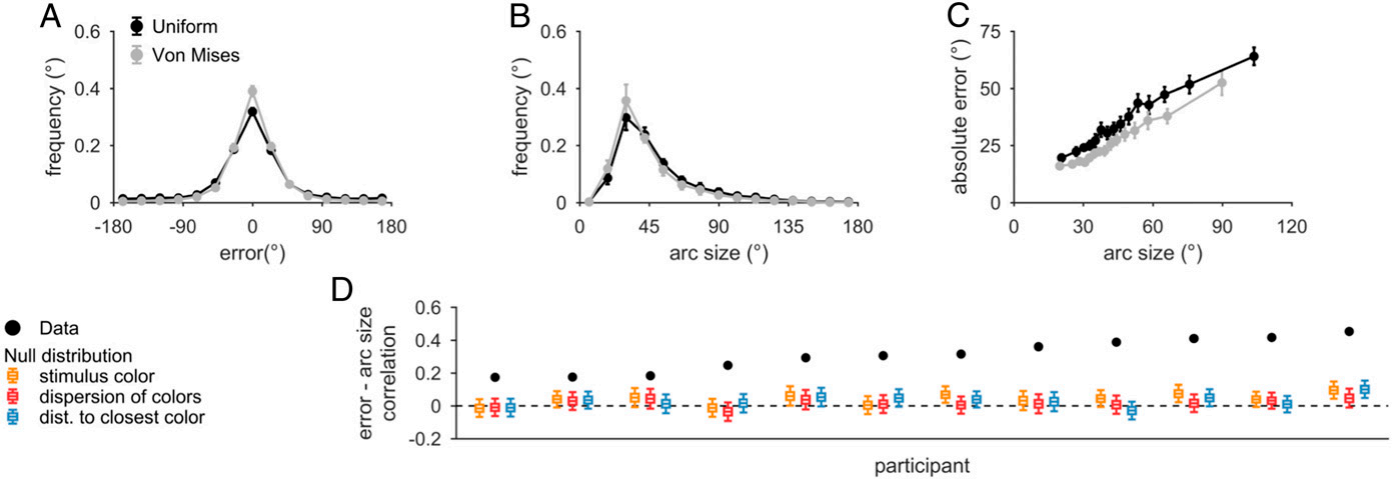
Arc size reports correlate with error and reflect trial-to-trial knowledge of memory quality. Behavioral data from uniform stimulus distribution trials (black) and von Mises stimulus distribution trials (blue) are shown. Here and elsewhere, error bars represent mean ± SEM across participants. (*A*) Histograms of circular estimation error in 24° bins. (*B*) Histograms of arc size in 12° bins. “Arc size” refers to one-half of the size of the reported arc. (*C*) Error and arc size, plotted in 15 quantile bins. For each participant, we separated the data into 15 quantiles of arc size and calculated the mean arc size and absolute estimation error per quantile. For each quantile, we then calculated the mean ± SEM of these averages across participants and plotted the point at the horizontal coordinate equal to the mean of the quantile centers. Error is correlated with arc size, suggesting trial-to-trial knowledge of memory quality. (*D*) Comparison of the observed error–arc size correlation coefficient to the null distribution expected if the correlation were driven solely by stimulus properties, dispersion of colors in a display, or distance to the most similar color in a display. The boxplot shows the 5th, 25th, 50th, 75th, and 95th percentiles. The observed correlation values fall far outside of the null distribution, indicating that they cannot be driven solely by stimulus color.

We first examined sessions with a uniform stimulus distribution. Participants produced two responses: errors (defined as the circular distance between stimulus and estimate, in degrees) and arc sizes (Fig. 2 *A* and *B*). Arc size is positively correlated with absolute estimation error on a single-trial level (“error–arc size correlation,” mean ± SEM across participants, *r*_s_ = 0.31 ± 0.029, *t*(11) = 10.2, *P* = 6.1 × 10^*−*7^) (Fig. 2*C*), such that riskier decisions (smaller arcs) were associated with smaller errors and vice versa. This shows that people know their trial-to-trial memory quality, consistent with the idea that WM represents memory uncertainty. However, accurate reports of memory quality do not necessarily imply that this uncertainty is stored in WM.

One possibility is that people use information about stimulus features as a proxy for uncertainty (27, 28). For instance, if an observer knows that they encode the color pink poorly, they could set a larger arc on trials with a pink stimulus. To test this, we simulated error–arc size correlations from the null hypothesis that correlation is caused by stimulus color (*SI Appendix*, section S1*B*). Observed correlations (*r*_s_ = 0.31 ± 0.029) are far larger than the mean simulated correlations (mean ± SEM, *r*_null_ = 0.010 ± 0.040; *P <* 10^*−*9^ for all participants). We repeated this analysis for two other potential confounding factors, the dispersion of the colors within a display (*r*_null_ = 0.016 ± 0.0068; *P <* 10^*−*9^ for all participants) (29) and the distance in color space from a probed color to the nearest nonprobed item (*r*_null_ = 0.0063 ± 0.0082; *P <* 10^*−*9^ for all participants) (30), as well as several others (*SI Appendix*, section S1*B*). All confounding factors predicted much lower correlations than those observed (Fig. 2*D*). This suggests that there is sizeable uncertainty due to internal processes (9), and that participants’ knowledge of memory quality is not explained by remembering stimulus properties (31).

The error–arc size correlation also could be explained by an observer’s limited knowledge of their uncertainty. For instance, even if there were no internal representation of uncertainty, an observer could know something about their memory quality simply by being aware of “lapse trials” in which they had low precision (e.g., blinking during stimulus presentation). In this case, the correlation would be caused by a mixture of normal trials (lower error, lower arc) and low-precision trials (higher error, higher arc) in which the observer knows they did not encode the stimulus. To explore this possibility, we built computational models of this task.

**Fig. 3.**
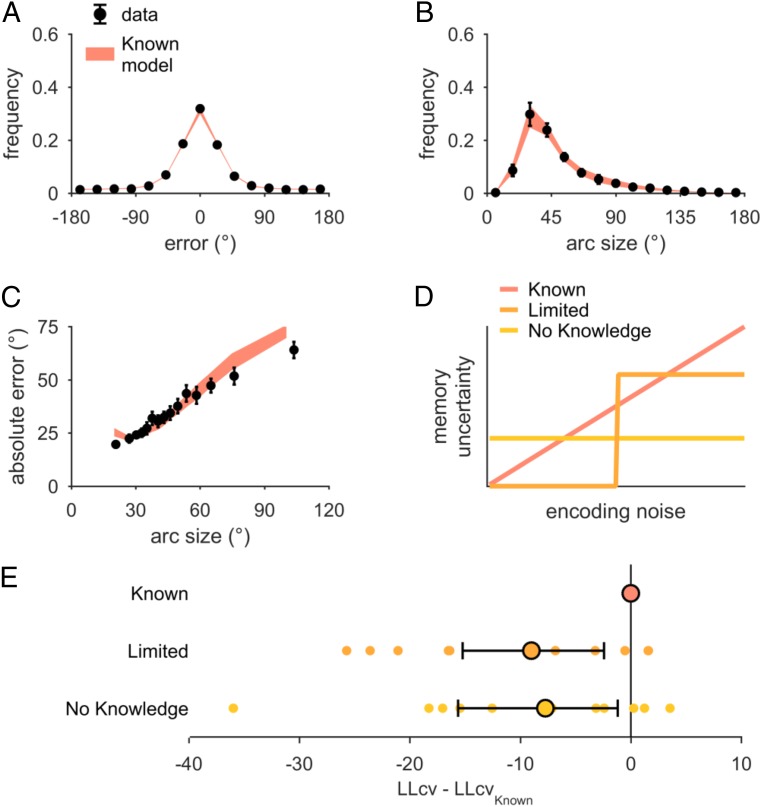
Model comparison, uniform stimulus distribution sessions. (*A*–*C*) Known model predictions, plotted with the same conventions as in Fig. 2. (*D*) Schematic representation of the relationship between encoding noise and memory uncertainty in tested models. Known, observers know their encoding precision perfectly; no knowledge, observers have no access to encoding precision and instead assume a fixed memory uncertainty (free parameter); limited, observers have limited knowledge of encoding precision and represent it as either 0 (when below a threshold) or a fixed value (both free parameters). (*E*) Model comparison using 10-fold LLcv. Dots indicate individual participant differences between each model and the known model. Circles and error bars represent mean and 95% bootstrapped confidence interval. Negative numbers represent a worse fit than the known model. The known model fits the best; results are consistent using the Akaike information criterion or the Bayes information criterion (*SI Appendix*, section S3*A*).

These models are variants of a Bayesian decision model built on top of a variable precision encoding model (Fig. 1*B*). In these models, stimuli are encoded as memories with a memory precision that varies across trials. We assume that the observer knows their encoding noise, represented as memory uncertainty, which they combine with their memory to build a posterior distribution. Stimulus estimates are noisily sampled from the posterior. Arc sizes are obtained by calculating the expected utility of each response given a memory and the reward rule and sampling from this distribution with (softmax) noise. We further assume that on each trial, the observer has a certain probability of “lapsing” and failing to encode the stimulus. In this case, the observer responds as if they had no memory information. This model (designated the Known model; six parameters) fits the data qualitatively well (Fig. 3 *A*–*C*); however, we tested modifications to the model to examine how memory uncertainty may contribute to arc reports. Models were compared using 10-fold cross-validated log-likelihood (LLcv) to account for differences in model complexity, with data reported as the mean LLcv difference from the best-fitting model followed by a bootstrapped 95% confidence interval in brackets. Importantly, this approach automatically penalizes overfitting, allowing more parsimonious models to perform better than flexible models.

We compare the Known model to alternatives in which encoding noise is not known. While we use the term “not known,” this model also includes the possibility that people know their uncertainty but do not use it in the response. The No Knowledge (seven parameters) model assumes that the observer does not know their encoding noise and uses a fixed uncertainty to set an arc. Although the observer does not know their encoding noise, they know when they are lapsing. This allows the model to predict a relationship between error and arc size without any knowledge of encoding precision. The Limited model (eight parameters) assumes that observers partially know their encoding noise; uncertainty is zero or a fixed value according to whether encoding noise exceeds a threshold (Fig. 3*D*). The Known model outperforms the No Knowledge (∆_LLcv_ = −7.8 [−15.1 −1.6]) and Limited (Δ_LLcv_ = −9.1 [−15.7 −2.7]) models (Fig. 3*E*). Furthermore, to confirm our assumption that precision is variable across trials, we compare the known model with a fixed precision model (32) in which encoding noise is always the same (*SI Appendix*, section S1*D*). This model performs poorly (∆_LLcv_ = −19.4 [−26.8 −13.0]), supporting previous results indicating that encoding precision is variable (9, 33). It is worth noting that there exist models between the Known and Limited models that were not tested, such as other forms of partial knowledge of uncertainty, which can be explored in future work. Nonetheless, our findings highlight that models without knowledge of encoding noise perform poorly, suggesting WM uncertainty as a causal factor of both estimation errors and arc responses.

### Von Mises Stimulus Distribution.

To test whether people could combine their WM uncertainty with prior information, we manipulated the stimulus distribution in two of the four experimental distributions to be a von Mises (circular normal) distribution, with a fixed width and a different mean for each participant. Repeating the previous analyses on the Von Mises stimulus distribution trials yielded consistent results (*SI Appendix*, section S3*B*). Participants were trained to learn the stimulus distribution (*SI Appendix*, section S1*A*), inducing prior beliefs about stimuli. Despite training, we found that participants underestimated the prior width (std = 60°) by reporting a width of 52.42°. Optimally incorporating these prior beliefs with memory uncertainty (using Bayes’ rule) would confer a performance advantage; for instance, if the color remembered were both highly uncertain and improbable given the believed stimulus distribution, then a more accurate estimate could be made by shifting the response toward more frequent colors (*SI Appendix*, section S2*A*). This predicts that responses incorporating prior information should be attracted toward the most frequent color.

**Fig. 4.**
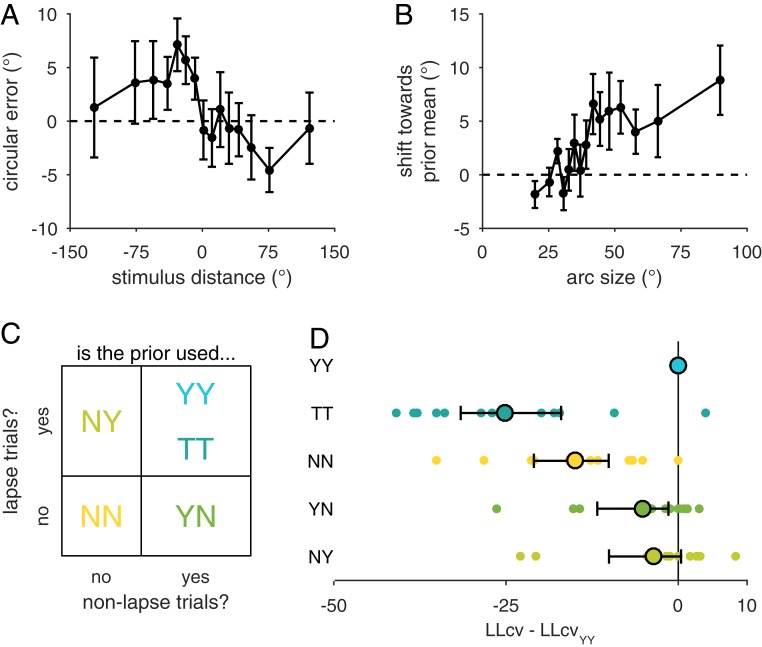
Prior information is incorporated into decisions. Shown are behavioral results from sessions with a von Mises stimulus distribution. Plots are binned in 15 quantiles of either stimulus distance from the prior or arc size, as in Fig. 2*C*. (*A*) Trial-averaged circular estimation error against stimulus distance from the most frequent color (prior mean). For stimuli counterclockwise (−) of the prior mean (0), errors were in the clockwise direction (+) and vice versa, showing that responses were shifted toward the prior mean. (*B*) Shift toward the prior mean is defined as the trial-averaged error toward (+) or away from (−) the prior mean. As reported arc size increases, responses shift more toward the prior mean. This suggests that memory uncertainty modulates the influence of prior information in the response. (*C*) Four prior use strategies are defined by whether observers use the prior (Y, yes; N, no) during nonlapse (first Y/N) and lapse trials (second Y/N). TT is a special case of YY in which an observer’s prior beliefs are the same as the true stimulus distribution. (*D*) Model comparison using summed 10-fold LLcv. Dots represent individual participant differences between each model and the YY model. Circles and error bars represent mean and 95% confidence interval. Negative numbers represent a worse fit compared with the YY model. Models that ignore the prior (NN) or use the true distribution (TT) perform poorly. The YY model is the best overall descriptor, but does not best describe all participants. Results are consistent using the Akaike information criterion or the Bayes information criterion (*SI Appendix*, section S3*A*).

Validating this, responses on the left (−) of the most frequent color (0) have rightward error (+) and vice versa (Fig. 4*A*). We quantify this by multiplying the error by the sign of the stimulus on that trial to obtain the directional shift toward (+) or away from (−) the prior. The mean shift toward the prior is positive, showing that stimulus estimates are attracted to the prior mean (mean shift = 3.1° ± 1.38, two-tailed Wilcoxon signed-rank test, *P* = 0.034) (11). Importantly, the amount of shift toward the prior mean is correlated with arc size, suggesting that people incorporate prior and memory information in proportion to their memory uncertainty [mean ± SEM, *r*_s_ = 0.080 ± 0.025, *t* (11) = 3.3, *P* = 0.0077] (Fig. 4*B*). This shift–arc size correlation is not explained by confounding factors (stimulus color, rs = 0.0034 ± 1.10; dispersion of items, *r*_*s*_ = 0.010 ± 0.0076; distance to closest item, *r_s_* = 0.0019 ± 0.0059). In addition, the correlation is not driven by differences in stimulus and prior color driving both shifts and arc size, as reported arc sizes were not influenced by whether this distance was small or large [split-half, *t* (11) = −0.21, *P* = 0.84]. Furthermore, the correlation of response error with the prior error (i.e., deviation of the prior relative to the stimulus) showed a stronger correlation (0.39) for less confident trials than for confident trials [0.22; *t* (11) = 3.72, *P* = 0.003] compared with a split-half analysis. This is direct evidence of a greater influence of the prior when reported uncertainty is high (34).

To understand how prior and memory information are incorporated, we take the model that best explains the uniform stimulus distribution data (Known model) and add prior beliefs to it, jointly fitting the model on uniform and von Mises stimulus distribution data. However, introducing a prior introduces two issues: how the observer combines prior and memory information and the observer’s prior beliefs about the stimulus.

An optimal observer would incorporate prior information into their decision. Specifically, they would use Bayes’ rule to combine the likelihood (and associated memory uncertainty) and the prior beliefs to obtain a posterior. However, observers may just ignore prior information. Furthermore, observers could use prior information with some kind of limited knowledge of memory uncertainty, for instance, the knowledge low-precision (lapse) trials. In the absence of memory information (lapse trial), the optimal thing for an observer to do would be to respond at the prior mean. In this way, the observer could avoid combining prior information and memory uncertainty by using prior information to respond when highly uncertain (lapsing). The observer then would still have a shift–arc size correlation. We represent this possibility by testing a model in which the observer uses prior information when lapsing but otherwise ignores the prior. Models are represented with two letters, the first indicating whether prior information is used in nonlapse trials (“Y” for yes, “N” for no) and the second indicating whether prior information is used in lapse trials (“Y” for yes, “N” for no) (Fig. 4*C*). In this framework, an observer who uses the prior with memory uncertainty in both lapse and nonlapse trials is designated YY, while an observer who ignores the prior is NN and an observer who only uses prior information during lapse trials is NY. We test the opposite model in which the observer combines prior information and memory uncertainty but responds randomly when lapsing (YN) for completion.

Furthermore, participants may have incorrect internal beliefs about the stimulus distribution (*SI Appendix*, section S2*B*). Our models allow for this possibility by allowing the prior width to be a free parameter, *κ*_*w*_, which is fitted to individual participants. To validate this, we tested a nested case of the YY model, where the observer knows the true stimulus distribution (TT; *κ*_*w*_ =1.422; 60°). The YY model fits better than TT (∆_LLcv_ = −25.3 [−31.9 −16.4]), suggesting that participants incorrectly learned or represented the stimulus distribution (inferred *κ_w_* = 0.3024; 111.7°).

Comparing the YY, YN, NY, and NN models, we see that the YY model has the most overall predictive power. The NN model performs poorly (∆_LLcv_ = −15.1 [−21.7 −10.0]) (Fig. 4*D*), suggesting that participant responses incorporate prior information. When looking at how prior information is incorporated, the YN and NY models perform only slightly worse than the YY model. While this could suggest that all models are inadequate descriptors of the data, the relatively large log-evidence differences across participants (Fig. 4*D*) suggest that perhaps different participants are best explained by different strategies of combining prior and memory information. Furthermore, we show that models generate different predictions for participant behavior using model recovery analyses (*SI Appendix*, section S3 *G* and *H*), which suggests potential individual differences in strategy.

To examine this, we applied a hierarchical model of the model evidence, Bayesian model selection (35), which assumes that participants use different models and estimates the frequency of models in the population. Fitting this hierarchical model returns model frequencies that favor the YY, NY, and YN models but do not predict that any model is best represented in the population (*SI Appendix*, section S2*C*). While it is important to note that this analysis has a small sample size, this hierarchical model describes the data better than any single model (Bayes factor = 5.74 × 10^4^), consistent with the suggestion that people may have different strategies of combining prior and memory information (*SI Appendix*, section S2*C*).

## Discussion

We know that people can accurately report the quality of their own memories (9, 10) and incorporate stimulus reliability into memory decisions (25, 26); however, this does not necessarily imply that WM represents uncertainty. We show that people’s knowledge of memory quality cannot be explained by stimulus display factors or limited knowledge of encoding precision, suggesting that WM represents memory uncertainty. Our modeling suggests that spontaneous fluctuations in memory uncertainty are common causes of both estimation errors and arc sizes in this task. Future models of WM and decision making will need to incorporate task-relevant WM uncertainty.

Furthermore, when provided with prior information about stimuli probabilities, the data are best described by models in which this information is combined with WM uncertainty, showing that WM uncertainty can be combined with other sources of information. We propose two strategies of doing this: using prior information only when highly uncertain or combining prior and memory uncertainty information with Bayes’ rule. Overall, the data are best described by a hierarchical model in which different people use different strategies of prior and memory uncertainty combination. This suggests that people are capable of combining prior information with memory uncertainty, although not all individuals may do so. Our model of prior use is one of a class of prior use strategies; there are many models in this family that we did not test, such as switching between likelihood and prior (36). Further work is needed to understand the exact strategies by which prior and memory uncertainty information are combined and how they may vary across individuals.

While we present evidence that WM contains a representation of memory uncertainty that can be combined with other sources of information, how this is implemented in the brain remains an open question. In our models, we assume that memories are probabilistic representations similar to von Mises distributions, yet there are many ways in which probabilistic information could be represented in WM. One implementation is probabilistic population coding, in which continuous probability distributions over feature space can be represented in a population of neurons (37–39). However, it is possible that a more limited form of probabilistic information is stored, such as several samples of a stimulus, from which a memory uncertainty can be inferred (40, 41). Determining how WM uncertainty is represented will require further work. Regardless of how the WM representation is structured, our findings highlight that current models of WM must be consistent with the idea that WM representations contain not only an estimate of the stimulus, but also a measure of memory uncertainty that can be used in subsequent decisions.

This work highlights how studying WM as a system integrated with decision making can yield new insights into the capacity and representational nature of WM. By requiring participants to reason about memory contents and manipulating the conditions under which these decisions were made, we show that WM representations contain a trial-level representation of probabilistic information, which is incorporated into subsequent decisions. This approach contrasts with that of many WM paradigms that aim to minimize decision elements to examine WM in isolation (i.e., delayed estimation). However, there is no such thing as a “pure” WM task—even simple paradigms involve reasoning about stored information. Furthermore, not only does WM involve decision making, but decisions in the real world often involve WM (e.g., crossing the street, picking up objects). This has been recognized in reinforcement learning and behavioral economics, in which task-relevant WM processes contribute to sequential value-based decisions (42–44), risky decisions (45, 46), and delay discounting (47, 48). Thus, we suggest that models and theory should focus on understanding memory decision stages instead of minimizing their contribution. Studying WM in tasks with more decision elements could reveal how the WM system functions realistically in parallel with other systems. Rather than treating WM storage capacity and decision making as separate fields of inquiry, we suggest that an attempt to bridge these fields together is necessary to understand WM as a full and integrated system.

## Materials and Methods

### Data and Code Availability.

All experimental and model code and other data related to this paper are available in Open Science Framework (49).

### Participants.

Twelve individuals (seven females; mean age, 21.75 y; range, 19 to 28 y) participated in four 40- to 60-min sessions of this experiment. Participants were recruited with flyers posted at New York University. Each participant provided informed consent, and this study conformed to the Declaration of Helsinki and was approved by the New York University Committee on Activities Involving Human Subjects.

### Stimuli.

The experiment was coded in MATLAB using PsychToolBox (50) and performed on a 40.9 × 32.5 cm Dell 1907FPC LCD monitor (1,290 × 1,025 resolution, 75-Hz refresh rate) at a viewing distance of 40 cm. Participants were shown colored dots (1.26° radius) of eccentricity 6.3°. Dot colors were sampled, with replacement, from a color space consisting of 360 equidistant colors using the CIE L*a*b (centered at *L* = 54, *a* = 18, and *b* = 8; radius of 59; available in mem_toolbox) (51). Stimulus and background luminance were roughly 30 ^cd^*/*m^2^ and 35 ^cd^*/*m^2^, respectively. In sessions 1 and 4, colors were sampled with equal probability (uniform distribution). In sessions 2 and 3, colors were drawn from a von Mises distribution with a random mean and fixed concentration parameter of 1.422 (60° circular SD). Participants were trained to learn these distributions (*SI Appendix*, section S1*A*).

### Trial Procedure.

On each trial, participants were shown four colored dots for 100 ms. After a delay of 1,000 ms, they reported (with a mouse and color wheel) the color of a randomly probed dot. After reporting their estimate, participants also made a rewarded decision. There was a 1,000-ms interval between trials.

### Rewarded Decision.

Participants made a rewarded decision, incentivized to reflect memory uncertainty. After reporting a stimulus estimate, participants adjusted the size of a symmetric “arc,” similar to a confidence interval, around the estimate to obtain a reward (18). If the true color was within the bounds of their arc, then the participant received points inversely and linearly related to the size of the arc (points = (180 − *A*)/*A* where *A* is the size in degrees of one-half the symmetric arc). If the true color was outside the arc, they received 0 points.

### Performance Incentivization.

Participants received points on a scale of 0 to 100 and completed the experiment either after achieving 18,000 points total or after 1 h had elapsed. Participants completed on average 1,206 (range, 904 to 1461) trials with 645 (range, 556 to 795) uniform stimulus distribution trials and 561 (range, 348 to 645) von Mises stimulus distribution trials. Participants were paid $10 per session and received a completion bonus of $10, as well as performance bonuses based on the summed points from three random trials (0 to $3).

### Statistics.

Correlations are Spearman correlations (mean and SEM reported). To evaluate whether the mean correlation is nonzero, we used two-tailed *t* tests on the Fisher-transformed coefficients. Bootstrapped confidence intervals used the bias-corrected and accelerated percentile method (10,000 samples).

### Model.

We assume that stimuli are encoded as noisy memories (represented by a von Mises distribution), with an encoding precision, κ, that varies between trials, represented by a gamma distribution (two free parameters) (9, 33, 52). We assume the observer knows their encoding precision, represented as memory uncertainty, κ*, the width of the likelihood. The likelihood is combined with prior beliefs using Bayes’ rule, generating a posterior distribution.

To obtain an estimate, we assume that observers sample from a noisy representation of the posterior where noise is represented by a free parameter exponent (53). We assume that the probability of an arc size is a softmax function of the expected utility, with a free temperature parameter representing decision noise. The expected utility is computed by multiplying the reward utility of an arc, *U*(hit), by the probability of getting that reward, *p*(hit). To account for different risk attitudes across individuals, we assume that the utility of a correct response is equal not to the amount of points obtained, but rather to the points transformed by raising them to a power, α, representing risk attitude (54). *p*(hit) is the integral of the posterior under the area covered by the arc. For computational tractability, we approximate this by assuming that in each trial, the observer integrates around the mean of their posterior instead of around their reported estimate, making the arc size dependent only on the width of the posterior (*SI Appendix*, section S2*C*). A lapse process is also implemented such that on every trial there is a probability, λ (free parameter), that the participant will have no information about the stimulus. In this case, the estimate is a random sample from a uniform distribution, and the arc size is determined by the expected utility of this uniform distribution. Complete equations are provided in *SI Appendix*, section S1*C*, and fitted parameters and predictions are available in *SI Appendix*, section S3*G*.

### Implementation and Validation.

Models are coded in MATLAB with tools from circ_toolbox (55). We compared models using *k*-fold (*k* = 10) LLcv, summed across folds. Since participants performed different numbers of trials, to limit the influence of participants with more trials than the group average, we averaged the per-trial LLcv and multiplied by the mean number of trials across participants. Each model has between five and nine free parameters that are fit to each participant using maximum likelihood estimation, optimized through 50 randomly started runs of the Bayesian adaptive direct search algorithm (56). Removing the utility nonlinearity, the decision noise or lapse rate significantly worsens model fit (*SI Appendix*, section S3*C*). Parameters and results are consistent across stimulus distributions and when the arc size is excluded from fitting (*SI Appendix*, section S3 *E* and *F*).

## Supplementary Material

Supplementary File
