# Supplementary material for: Humans incorporate trial-to-trial working memory uncertainty into rewarded decisions
Source: Proc Natl Acad Sci U S A. 2020 Mar 30;117(15):8391–7. doi: 10.1073/pnas.1918143117 (PMC7165478; doi:10.1073/pnas.1918143117)
Supplement: Supplementary File [file pnas.1918143117.sapp.pdf]

PNAS
www.pnas.org

Supplementary Information for

Humans incorporate trial-to-trial working memory uncertainty into rewarded decisions

Maija Honig and Wei Ji Ma and Daryl Fougnie

Daryl Fougnie.
E-mail: darylfougnie@nyu.edu

This PDF file includes:

Figs. S1 to S15
Tables S1 to S3
References for SI reference citations

www.pnas.org/cgi/doi/10.1073/pnas.1918143117

Supplementary plots, analysis and model validation. Unless otherwise stated, both data-based and model-based analyses are performed on uniform stimulus distribution data, error bars are one standard error of mean, model-based analyses are performed using MLE parameters fit to each participant, and p values are from a Wilcoxon signed rank test (df=11).

Contents

1 Supplementary methods **3**
- A Distribution training . . . 3
- B Confounding variable analysis . . . 3
- C Model equations . . . 4
- D Modifications of the model . . . 6
- E Expected utility . . . 6

2 Supplementary results **8**
- A Additional models of prior information use . . . 8
- B People report different dynamic ranges of arc size . . . 10
- C Performance improves by incorporating prior beliefs . . . 11
- D No evidence for strong trial dependencies . . . 12
- E No evidence for systematic color biases . . . 12

3 Model validation **13**
- A Model comparison with AIC and BIC . . . 13
- B Model comparison including von Mises and uniform distribution data . . . 14
- C Our approximation to $p(\text{hit}|A, \hat{s}, x)$ is good . . . 14
- D Model parameters are all necessary . . . 15
- E Parameters are consistent for individuals . . . 16
- F Delayed estimation model fits (no arc response) . . . 16
- G Model distinguishability: recovery simulations . . . 17
- H Model distinguishability: the KL divergence . . . 17
- I The expected distribution of the KL divergence . . . 19

4 Misc. **20**
- A Table of best model parameters . . . 20
- B Model fits . . . 21

1. Supplementary methods

A. Distribution training.

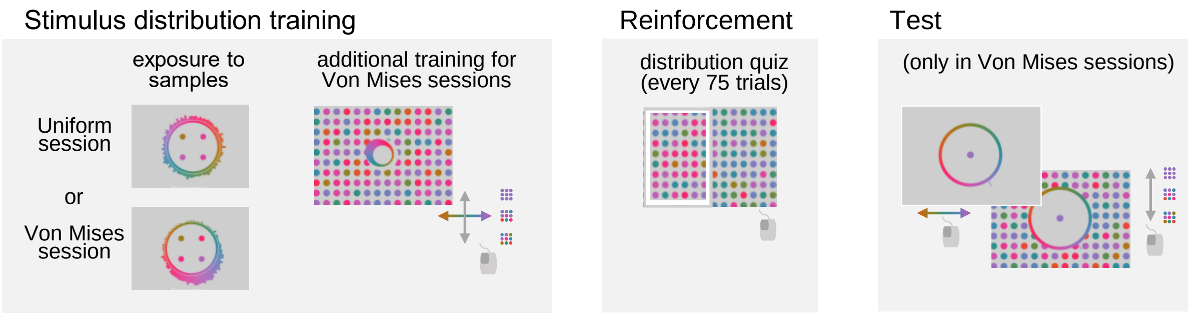

Fig. S1. Diagram of stimulus distribution training (see below).

Participants were given extensive training to learn the stimulus distribution of each session. Participants were shown a movie consisting of 1000 samples of stimuli drawn from the session's distribution. To provide a further visualization, samples were used to build a histogram of the session's color distribution Figure 1b). In von Mises sessions, during training participants further performed a "match assessment" (Figure 1b), where they used the mouse to simultaneously adjust the mean (left-right mouse position) and spread (up-down mouse position) of a sample of colored dots shown on screen to be representative of the learned distribution. Participants had to repeat this "match" assessment until they created a dot distribution close to the true distribution 3 consecutive times (mean error $<$ 20 degrees, circular standard deviation error $<$ 20 degrees).

Throughout the study, every 75 trials, participants were given a set of six binary choice questions about the session's color frequencies to reinforce knowledge of the stimulus distribution (Figure1b). Participants saw a bisected screen of two sets of dots and had to click on the set which best reflected the session's color distribution. In order to continue the experiment, participants had to answer all six of these questions correctly. Participants received feedback and were given unlimited attempts to complete the questions. The foil distributions were sligHly altered by shifting the mean color 60 degrees clockwise or counterclockwise (randomly) and the standard deviation by 30 degrees up or down in six combinations (narrow, wide, uniform, shifted, narrow-shifted, wide-shifted). The order of these foils was randomized in the questions.

To check whether participants learned the color frequencies, at the end of von Mises sessions, we had them report about their prior beliefs by generating a set of dots that matched the color distribution (similar to the earlier "match" assessment). They did this by using the mouse (left-right) to first select the most frequent color (mean of the distribution), and then moving the mouse (up-down) to set the spread of colors (circular standard deviation) (Figure 1b). Participants made three consecutive judgments and were awarded a performance bonus of 1 dollar if they reported the color frequencies accurately at least once (mean error $<$ 20 degrees, circular standard deviation error $<$ 20 degrees).

B. Confounding variable analysis.

We tested whether the observed correlation between absolute error and arc size (r_{obs}) was caused by both variables being correlated with a confounding variable such as stimulus color. To do this, we simulated data under the null hypothesis that the correlation is entirely caused by the stimulus color through shuffling our data. We sorted trials by stimulus color into groups of 5 trials with equal or similar stimulus color. Within each group, we then shuffled the arc size values uniquely such that each arc size was now associated with a different absolute error than on the trial it came from, but a similar stimulus color. This created a dataset where the relationship between arc size and error was solely dependent on the value of the stimulus color. We then correlated the shuffled arc sizes with the absolute error and obtained a value of r_{null} (similar to a semi-partial correlation). We repeated this 10,000 times for each participant to obtain the distribution of r_{null} and used this distribution to compute the probability of the observed r_{obs} per participant as well as the mean r_{null}, $\bar{r}_{\text{null}}$. In addition to stimulus color, we apply the exact same analysis to the Hs of the circular variance of the four displayed items on each trial, absolute color distance from the stimulus to the most confusable (closest in color space) item, the stimulus location, mean display color, and trial number.

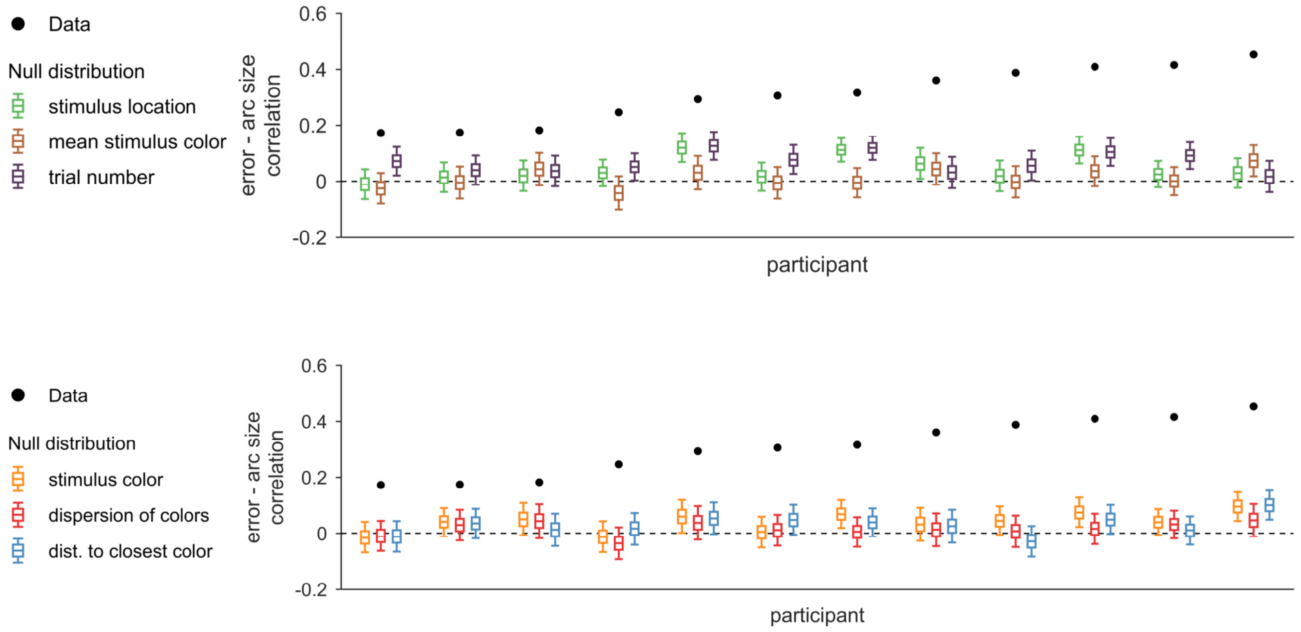

Fig. S2. yyy

confounding variable	mean r	s.e.m. r
stimulus color	0.0402	0.0098
circular variance	0.0164	0.0068
dist. to most confusable	0.0293	0.0097
stimulus location	0.0459	0.0097
mean display color	0.0121	0.0129
trial number	0.0689	0.0103
data correlation	0.3099	0.0286

C. Model equations.

We model this task using a Bayesian decision model built on top of a variable-precision encoding model. We assume that on each trial, the observer encodes the stimulus s as a noisy memory x. We assume that x follows a von Mises distribution with mean s and precision (concentration) parameter κ. Variable precision means that κ itself varies from trial to trial; following previous work (1–3), we assume it obeys a gamma distribution parameterized by its mean ($\bar{\kappa}$) and shape parameter (ϕ).

$$p(x|s,\kappa) = \frac{1}{2\pi I_0(\kappa)} e^{\kappa \cos(x-s)}$$

Variable precision means that κ itself varies from trial to trial; following previous work (1–3), we assume it obeys a gamma distribution parameterized by its mean ($\bar{\kappa}$) and shape parameter (ϕ),

$$p(\kappa|\bar{\kappa},\phi) = \frac{1}{\Gamma(\phi)(\frac{\bar{\kappa}}{\phi})^{\phi}} x^{\phi-1} e^{x(\frac{\phi}{\bar{\kappa}})}$$

The model observer stores on each trial a representation of this precision κ as certainty κ^*, where we assume for the moment that $\kappa^* = \kappa$. The observer combines the memory x and associated memory uncertainty κ^* (von Mises) with their prior beliefs (von Mises) using Bayes' rule to compute a posterior $p(s|x)$. Since the product of two von Mises distributions is again a von Mises distribution, the posterior is a von Mises distribution with an analytically defined mean μ_{pos} and concentration κ_{pos} (4):

$$\begin{aligned} p(s|x) &= p(s)p(x|s,\kappa^*) = \text{vonMises}(\mu_{\text{pos}}, \kappa_{\text{pos}}) \\ \kappa_{\text{pos}} &= \sqrt{\kappa_w^2 + \kappa^2 + 2\kappa_w \kappa \cos(0-x)} \\ \mu_{\text{pos}} &= \text{atan2}(\kappa_w \sin(\mu) + \kappa \sin(x), \kappa_w \cos(\mu) + \kappa \cos(x)) \end{aligned}$$

Where μ and κ_w represent the mean and concentration parameter of the prior and x and κ represent the mean and concentration parameter of the likelihood function. We assume that the observer bases their prior beliefs on the learned stimulus distribution in the current session. Thus, we assume that in sessions 1 and 4, the prior was a uniform distribution, while in sessions 2 and 3, it was a von Mises distribution centered at the stimulus distribution mean; we allow its concentration parameter to be a free parameter, κ_w.

$$p(s|\kappa_w) = \text{Uniform}(-180, 180) \text{ or vonMises}(s; \mu, \kappa_w)$$

To obtain an estimate of the remembered color, $\hat{s}$, the observer samples from the posterior raised to a power ν, which represents decision noise (5).

$$p(\hat{s}, x|\kappa^*, \nu) = \frac{p(s|x)^\nu}{\int p(s|x)^\nu \mathrm{d}s}$$

We assume that the probability that the the observer sets arc size A is a softmax function of the expected utility, with inverse temperature β,

$$p(A|\alpha, \beta, x, \kappa^*) = \frac{e^{\beta \mathrm{EU}(A|\alpha, x, \kappa^*)}}{\int e^{\beta \mathrm{EU}(A|\alpha, x, \kappa^*)} \mathrm{d}A}$$

The expected utility is computed by multiplying the reward utility of an arc, $U(\text{hit})$, by the probability of getting that reward, $p(\text{hit})$.

$$\mathrm{EU}(A, p(s|x), \alpha) = U(\text{hit}|A, \alpha) p(\text{hit}|A)$$

We assume that the utility of a correct response is not equal to the amount of points obtained ($R(A)$, a linear function), but the points transformed by raised to a power α, representing risk attitude (6).

$$R(A) = \frac{180 - A}{180}$$
$$U(\text{hit}|A, \alpha) = R(A)^\alpha$$

The probability of a hit is the integral of the posterior under the area covered by the arc. This is a von Mises distribution with parameters μ_{pos} and κ_{pos}

$$p(\text{hit}|A) = \int_{\hat{s}-A}^{\hat{s}+A} p(s|x) \mathrm{d}s = \int_{\hat{s}-A}^{\hat{s}+A} \text{vonMises}(s; \mu_{\text{pos}}, \kappa_{\text{pos}}) \mathrm{d}s$$

This is computationally demanding, since $p(\text{hit})$ depends on the stimulus estimate $\hat{s}$, arc size A, mean of the posterior μ_{pos}, and width of the posterior κ_{pos}. To simplify, we can rewrite this expression, from which it is apparent that $p(\text{hit})$ is not a function of $\hat{s}$ and μ_{pos}, but the difference between them $\hat{s} - \mu_{\text{pos}}$

$$p(\text{hit}|A) = \int_{\hat{s}-\mu_{\text{pos}}-A}^{\hat{s}-\mu_{\text{pos}}+A} \text{vonMises}(s; 0, \kappa_{\text{pos}}) \mathrm{d}s$$

If we make the approximation that $E[|\hat{s} - \mu_{\text{pos}}|]$ is small ($\hat{s} - \mu_{\text{pos}} \approx 0$):

$$p(\text{hit}|A, p(s|x)) \approx \int_{0-A}^{0+A} \text{vonMises}(s; 0, \kappa_{\text{pos}}) \mathrm{d}s \approx 2 \int_{0}^{A} \text{vonMises}(s; 0, \kappa_{\text{pos}}) \mathrm{d}s$$

This removes the estimate from the integral and causing $p(\text{hit})$ to be only dependent on A and κ_{pos}. This approximation has the effect of mildly underestimating arc sizes (see Supplementary material). However, since even after approximation, computing the von Mises integrals is computationally heavy we speed this up by precomputing a table of $p(\text{hit})$ for 180 A values and 1000 κ_{pos} values and linearly interpolating.

Furthermore, a lapse process is implemented such that on every trial there is a probability λ that the participant will have no information about the stimulus (for example due to a failure to encode the stimulus (7)). In this situation the observer will report a random estimate and an arc size according to the expected utility function associated with a flat posterior.

$$p(\hat{s}, A|x, \kappa) = p(\hat{s}|x, \kappa)p(A|x, \kappa)(\lambda)+ \\ (1-\lambda)p(\hat{s}|x, \kappa=0)p(A|x, \kappa=0)$$

For the joint probability of $\hat{s}$ and A given the stimulus s and parameters $\theta = [\alpha, \beta, \bar{\kappa}, \phi, \nu, \lambda, \kappa_w]$, we obtain

$$p(\hat{s}, A|s, \theta) = \iint p(\hat{s}|x, \kappa)p(A|\theta, x, \kappa)p(x|s, \kappa)p(\kappa|\theta)\mathrm{d}x\mathrm{d}\kappa$$

D. Modifications of the model.

To test the assumptions of this model, we modify the base model (**vp-known / vp-k**, 6 parameters) in several ways:

- **VP-known (6p)**: No modifications
  Base model, a model in which the observer encodes stimuli with variable encoding precision (parameter κ) and represents this encoding precision as memory uncertainty (κ^*).
- **FP-known (5p)**: No variable precision
  A model in which people do not have internal fluctuations encoding precision, but always encode stimuli with a single fixed precision ($\kappa = \kappa_{\text{fixed}}$). In this model, knowledge of memory quality is limited to knowledge of zero-precision trials ("lapses").
- **VP-inaccessible (7p)**: No knowledge of memory uncertainty
  A model in which people have internal fluctuations in encoding precision, but have no knowledge of these fluctuations and assume their memory uncertainty is a fixed value ($\kappa^* = \kappa_{\text{assumed}}$). In this model, knowledge of memory quality is limited to knowledge of zero-precision trials ("lapses"). κ_{assumed} is a free parameter.
- **VP-discrete access (8p)**: Limited knowledge of memory uncertainty
  A model in which people have limited knowledge of their memory uncertainty: when it is above a threshold $\kappa_{\text{threshold}}$ they assume it is a fixed value κ_{assumed}, while if it is below the threshold they assume it is zero. κ_{assumed} and $\kappa_{\text{threshold}}$ are free parameters.
  $$\kappa^* = \begin{cases} \kappa_{\text{assumed}} & \kappa \geq \kappa_{\text{threshold}} \\ 0 & \text{otherwise} \end{cases}$$

When the stimulus distribution is von Mises, to test how people incorporate prior and memory uncertainty information we test several different mechanisms of prior combination. Since an assessment of people's prior knowledge revealed misconceptions about its width, the prior width in these models is a free parameter κ_w unless otherwise stated. Models are named by their use of the prior during the task (first letter) or when lapsing (second letter) represented by the letters "Y" (*yes* prior use, prior width is a free parameter κ_w), "N" (*no* prior use), or "T" (using the *true* stimulus distribution).

- **TT (6p)**: Always use the prior, perfect knowledge of prior
  Memory and prior are combined with Bayes' rule. Prior width is not a free parameter but is fixed at the actual value ($\kappa_w = 1.422$).
- **YY (7p)**: Always use the prior
  Memory and prior are combined with Bayes' rule.
- **YN (7p)**: Use the prior, except when precision is zero
  Memory and prior are combined with Bayes' rule, except when precision = 0. In that case, the observer does not use the prior and responds with a random estimate and an arc size based on a uniform posterior (random guess).
- **NY (7p)**: Only use the prior when precision is zero
  When responding from memory, participants ignore the prior. However, when precision = 0, the observer uses the prior to make an informed guess of the estimate, and sets an arc size based on the width of the prior.
- **NN (6p)**: Never use the prior or has a flat prior
  The prior is never used ($\kappa_w = 0$).

E. Expected utility.

Our models assume that the expected utility of an arc sizes is the utility of the arc response multiplied by the probability of receiving reward ("probability of hit"). Thus the optimal arc size is determined by the maximum of this function. This is analytically difficult due to the fact that the hit probability is a Von Mises integral, which makes differentiation difficult. Furthermore we assume that the reward utility is the reward raised to a power representing risk attitude (alpha), which means the optimal arc size as a function of uncertainty changes depending on the agent's risk attitude.

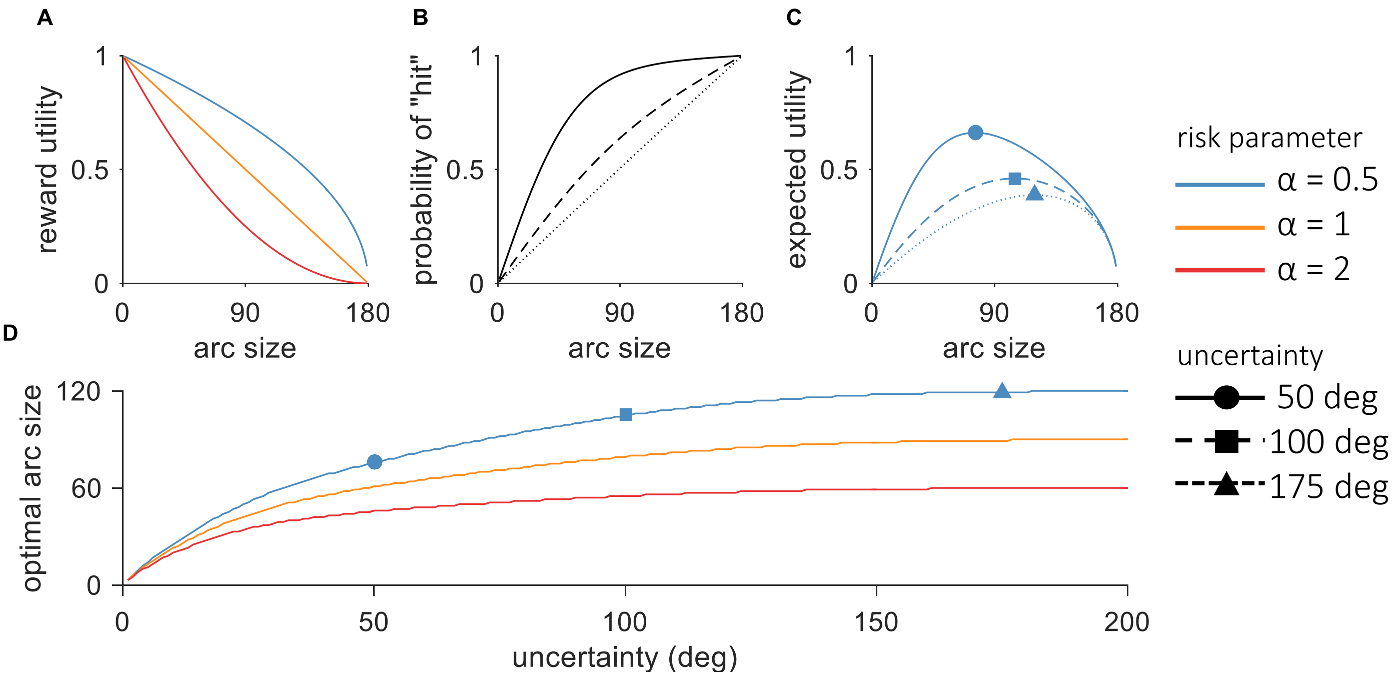

Fig. S3. Visual depiction of expected utility A diagram of the optimal arc size as a function of uncertainty, and how this is calculated. The expected utility of an arc size is the utility multiplied by the probability of obtaining reward (probability of hit). Furthermore, we incorporate risk attitudes into this calculation by having the utility be the reward raised to a power (alpha). Plotted (A) are the utility-arc size curves for three possible risk attitudes, risk averse (alpha=.5), risk neutral (alpha=1), and risk prone (alpha=2). The reward utility is multiplied by the hit probability, a von mises distribution integrated over the size of the arc (see methods). Thus the hit probability increases as uncertainty decreases. By multiplying these two distributions (C), we obtain the expected utility of an arc response. The optimal arc size for a given uncertainty is the arc size which maximizes the expected utility (blue dots). The optimal arc size (D) increases monotonically with uncertainty, and begins to asymptote as uncertainty becomes high. Noticeably, a risk prone attitude (red) promotes smaller arc sizes than a risk averse attitude (blue).

2. Supplementary results

A. Additional models of prior information use.

The YY model also captures the qualitative trends of the data (see Supplemental material), including the correlation between response shift towards the prior and arc size. However, this correlation may not result from the combination of prior information and memory uncertainty, but from an observer who uses prior information only when highly uncertain (i.e. a lapse trial). This would generate a mixture of high uncertainty high shift trials and low-uncertainty zero shift trials. To test this, we test models in which participants exhibit different prior-use behavior in lapse trials compared to non-lapse trials. On a lapse trial, where the observer has no information about the stimulus, a prior-using observer would respond at the mean of the prior (since the likelihood distribution is flat), while a non prior-using observer would respond with a random (uniform) guess when lapsing. We denote models with two letters, the first indicates whether prior information is used (“Y"=yes, “N"=no) during non-lapse trials, while the second indicates whether prior information is used while lapsing (Figure SX). In this framework the YY and NN models are the same as above (respectively using, and not using prior information). However, we introduce a new model, in which the observer only uses prior information when lapsing (**NY**). This would generate a mixture of non-lapse trials with no prior shift, and lapse trials with a large amount of prior shift, creating a correlation between prior shift and arc size. For completeness, we also test the opposite model, where participants use prior information during non-lapse trials, but respond from a flat likelihood distribution when lapsing (**YN**).

We equipped the two new models (YN, NY) with a free parameter representing the concentration parameter of the prior (as in the YY model), and fit them jointly on both uniform and von Mises stimulus distribution trials. While the YY model still has the most predictive power, we find that both the NY ($\Delta_{\mathrm{LLcv}} = -3.4, [-10.2\ 0.6]$) and YN ($\Delta_{\mathrm{LLcv}} = -5.5, [-12.4 - 1.6]$) models fit the data almost as well (Figure SX). However, despite small mean differences in model evidence, there is high variance in model evidence differences across participants. Even the model with the most predictive power (YY) does not describe many participants best, suggesting that people could have different strategies of prior use, better explained by different models.

To examine whether different people could have different strategies of using prior information, we assume that our participants do not all use the same model, and examine an alternative, hierarchical, model of how participants could be distributed using Bayesian Model Selection (BMS) (8). This is a hierarchical model which treats models as random variables (“random effects") and estimates the parameters of a Dirichlet distribution describing the probability of each model given the log-evidences (in our case the summed 10-fold cross validated log-likelihood) for each model and participant. By applying this hierarchical model we can obtain those Dirichlet parameters and thus the probability of each model occurring in our participant population (Figure SX, as well as the protected exceedance probability, the probability that a given model is the most likely in the population above and beyond chance (9). Overall most (62 %) of participants are estimated to come from models in which they use the prior with memory information (YY: 0.29, YN: 0.22, TT: 0.11) while the other participants either do not use the prior (NN: 0.06) or use the prior without memory uncertainty (NY: 0.32). The protected exceedance probability is low even for the maximally represented model NY (0.31, Figure SX, see Supplementary materials), suggesting there is no dominating model among our participants.

This suggests that people can incorporate prior information and memory uncertainty in simple decisions, though not all people may do so. To confirm that participants use different strategies, we compare the probability of participants all being from the same model (“fixed effects hypothesis”) to the probability of participants being from different models (“random effects hypothesis”, see Methods). The random effects hypothesis is far more probable than fixed effects (random/fixed odds-ratio $= 5.74 \cdot 10^4$), suggesting that it is more likely that strategies of prior use vary across individuals, then that they use any single strategy captured by our models. Regardless of the underlying algorithm, we suggest that individuals can combine prior information with memory uncertainty. Further work will be needed to understand how prior information and memory uncertainty are combined.

Fixed/Random odds ratio.

To check whether there is evidence for participants using different models, we calculate the ratio of the probability of all participants following the same model (H0, fixed effects) against the probability of participants following different models (H1, random effects) given the data y and models $m_1 ... m_K$ where K is the number of models. $p(y_n|m_k)$ is the likelihood of the data for a participant n given a model m_k.

$$\text{random odds ratio} = \frac{p(y|H_0)}{p(y|H_1)}$$

$p(y|H_0)$ is the probability of data being from a single model m_k, multiplied by the prior probability of that model and summed for all possible models

$$p(y|H_0) = \sum_k p(y|m_k)p(m_k)$$

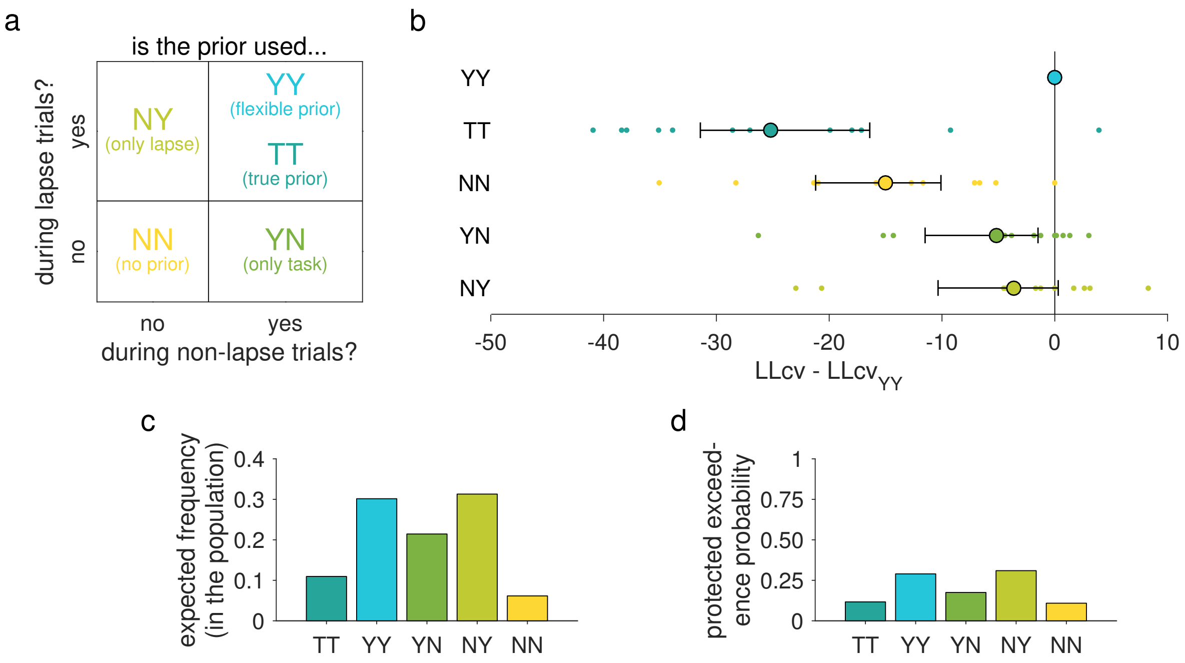

Fig. S4. Comparison of prior use models. Comparison of five models that differ in the way by which prior information is used. **(a)** We define each prior-use strategy by whether ("Y") or not ("N") observers use the prior during non-lapse (first Y or N) and lapse trials (second Y or N). A special case of the YY strategy is one in which the observers use the true stimulus distribution as their prior (TT). **(b)** Model comparison using summed 10-fold cross-validated log-likelihood (LLcv). Dots: individual-participant differences between each model and the YY model. Circles and error bars: mean and 95 % confidence interval. Negative numbers represent a worse fit than the YY model. Models which ignore the prior (NN) or use the true prior width (TT) perform poorly. The YY model describes the data best overall, but does not describe each participant best. Results are consistent when using AIC or BIC (see Supplementary material). **(c)** Expected frequencies of the models in the population, obtained using Bayesian Model Selection. Overall, most participants are expected to use the prior in some way (YY,YN,NY,TT), but are split between participants who combine the prior with memory uncertainty (YY,YN,TT) and those who do not (NY). This suggests that the way that individuals use prior information in this task varies. **(d)** Protected exceedance probabilities of model obtained from Bayesian Model Selection. This is the probability that a given model is the most likely model in the population, which is low even for the most likely model (NY).

The probability of data y being from a model m_k is the product of the model likelihood $p(y_n|m_k)$ for each participant n, which is the sum of the log-likelihoods.

$$p(y|m_k) = \prod_n p(y_n|m_k) = \exp \sum_n \log p(y_n|m_k)$$

Assuming a uniform prior over models:

$$p(m_k) = \frac{1}{K}$$

This gives us the full equation for $p(y|H_0)$:

$$p(y|m_i) = \frac{1}{K} \sum_k \exp \sum_n \log p(y_n|m_k)$$

For $p(m|H_1)$, we use the probability of the random-effects group BMS model (9):

$$p(y|H_1) = p(y|\text{BMS})$$

The fixed/random odds ratio reflects the relative probabilities of H_0 and H_1, large values reflect model evidence consistent with participants all being from the same model while small values are evidence that participants use different models.

B. People report different dynamic ranges of arc size.

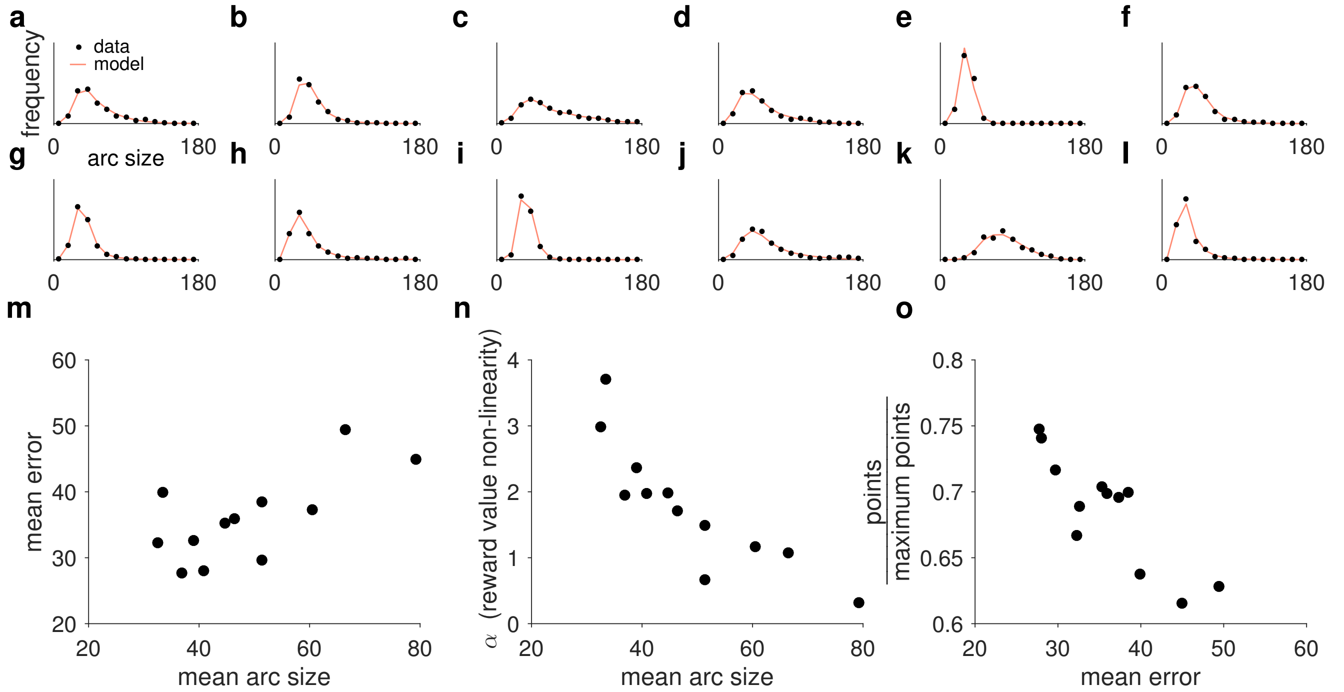

Fig. S5. (a-l) Histogram of arc responses in uniform stimulus distribution trials for individual participants, binned in increments of 12 degrees (black dots). Participants report varying ranges of arc sizes, and the distributions are well explained by the VP-known model (red line). **(m)** Mean error and mean arc size for each participant are correlated across participants ($r_s = 0.59, p = 0.048$), suggesting that across participants, arc sizes reflect task performance. **(n)** A parameter in our models , α, relates utility to reward, Utility(A) = Reward$(A)^\alpha$. Thus, $\alpha > 1$ represents a risk-seeking attitude, while $\alpha < 1$ represents a risk-averse attitude. In the VP-known model to uniform stimulus distribution data tend to have $\alpha > 1$ (mean and s.e.m = 1.78 ± 0.275, Wilcoxon signed rank test, $p = 0.012$). Supporting that α reflects risk attitude, across participants, α is inversely correlated with mean arc size ($r_s - 0.90, p < 10^{-16}$). **(o)** To examine how good participants are at obtaining points by reporting arc sizes, we examined their ability to report points. However, we cannot look at the mean points obtained for each participant, since the amount of points obtained is a function of a participants errors as well as their arc sizes. To account for this, we calculate the mean ability to obtain points as the ratio of points obtained on a trial (from their error and reported arc size), divided by the maximum amount of points that could have been obtained on that trial given the error (this is the points obtained from an arc size equal to the trial error+1). Individual differences in the ability to report points are inversely correlated with error ($r_s = 0.80, p = 0.0032$), suggesting that memory quality and the ability to know one's memory quality are related.

C. Performance improves by incorporating prior beliefs.

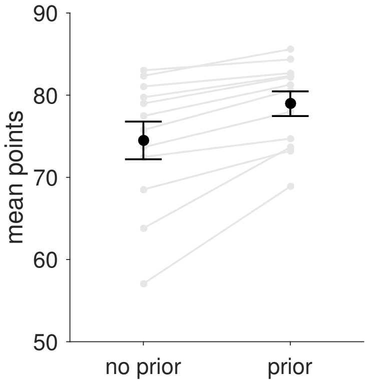

Fig. S6. To show that participants were sufficiently incentivized to use prior information, we used simulations to quantify the potential performance improvement from using prior information. Using the parameters (VP-known model) fitted to participants' uniform stimulus distribution sessions, we simulated data using a model where prior and memory information are optimally incorporated (TT model, "prior") and a model in which prior information is not incorporated into the response (NN model, "no prior"). Black error bars are the mean and s.e.m. across participants, while the gray lines are individual participant simulations. Adding a prior leads to an increase in average points for all participants (mean and s.e.m. $\text{points}_{\text{VM}} - \text{points}_{\text{Unif}} = 4.47 \pm 0.93$, Wilcoxon signed rank test $p = 4.8 \cdot 10^{-4}$). Accumulated across a session, participants would be able to finish several minutes earlier as well as potentially earn a higher performance bonus, incentivizing them to use prior information.

D. No evidence for strong trial dependencies.

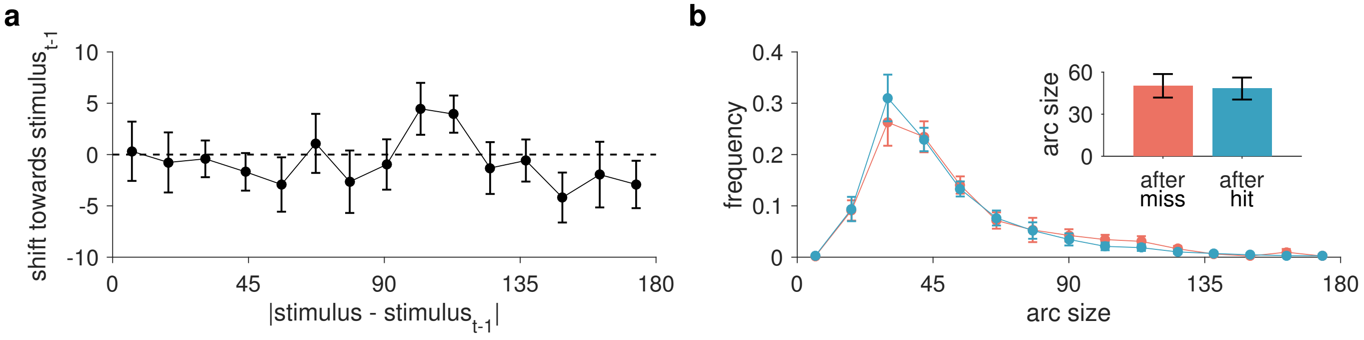

Fig. S7. **(a)** Examining the circular error (15 quantile bins averaged across participants) towards or away from the previous trial stimulus the data, we find no significant shift (+) or repulsion (-) from the probed stimulus of the previous trial (mean and s.e.m. $= -0.60 \pm 0.61, p = 0.233$). **(b)** Arc size reports (binned in increments of 12 degrees, averaged across participants) are sligHly biased by whether the previous trial was a hit (rewarded) or miss (no reward) trial with people reporting smaller arc sizes after a hit and larger after a miss (median arc after miss trial - median arc after hit$= -2.12 \pm 1.58, p = 0.016$). However, the effect size is extremely small (4.37% of the size of the mean reported arc).

E. No evidence for systematic color biases.

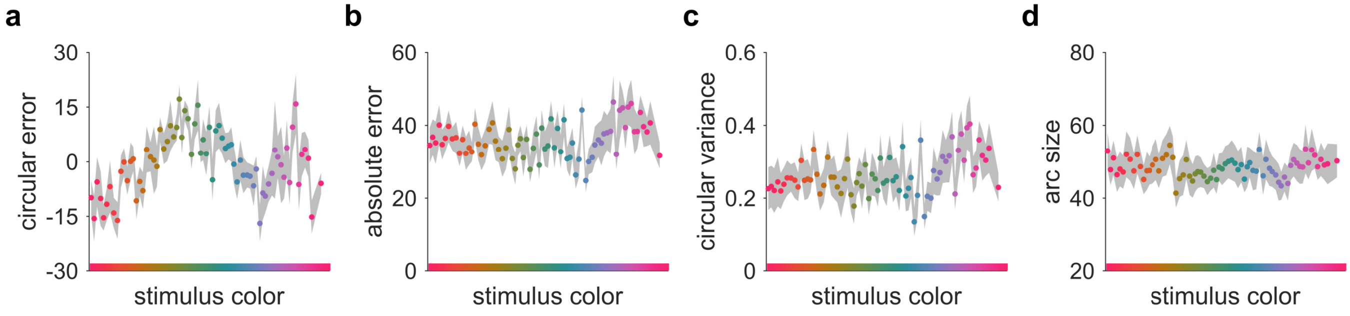

Fig. S8. Circular and absolute error, variance, and arc size as a function of stimulus color. Binned in increments of 5 degrees with mean (dots) and s.e.m. (grey area) plotted. **(a)** Participants' circular errors may be systematically attracted to dark blue and orange colors (they have rightwards error when the stimulus was to the left and leftwards error when it was to the right). **(b)** Absolute error of responses relatively is constant across colors, but sligHly higher for pink than other colors. **(c)** Circular variance of responses relatively is constant across colors, but sligHly higher for pink than other colors. **(d)** Arc size reports do not vary as a function of stimulus color. Overall there is no evidence that arc size is systematically biased by stimulus color, though there are some stimulus colors which are associated with higher estimate error and variability.

3. Model validation

A. Model comparison with AIC and BIC.

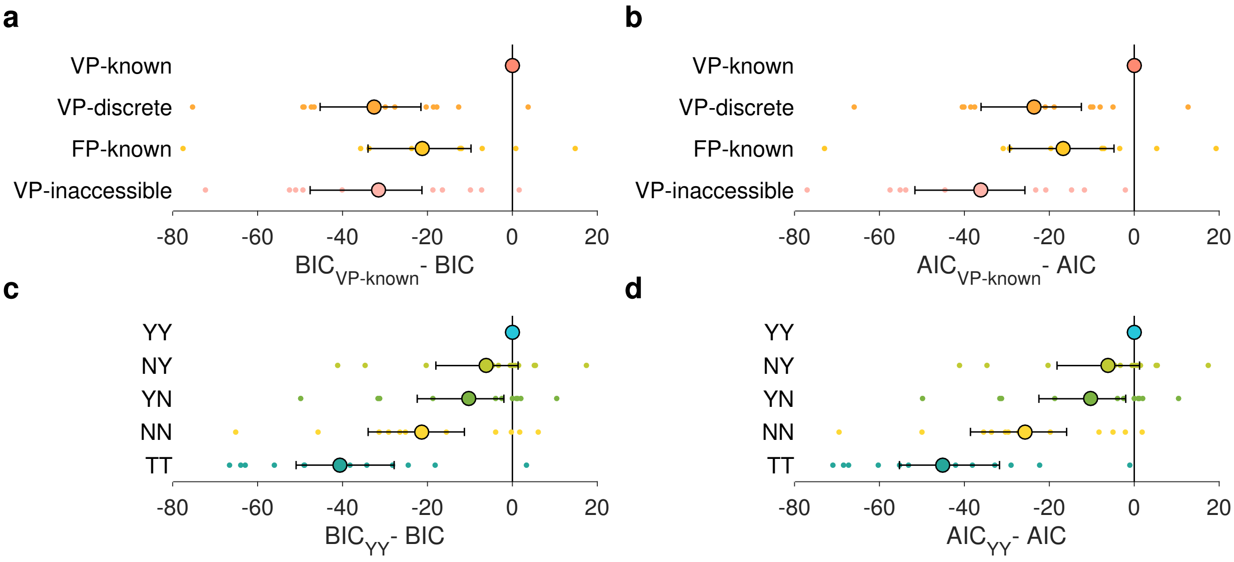

Fig. S9. Model comparison results using BIC or AIC are consistent with those using 10-fold cross-validated log-likelihood in the main text. **(a-b)** corresponds to main Figure 4, The VP-known model describes the uniform stimulus distribution best. **(c-d)** corresponds to main Figure 6. Models which incorporate prior information (with a free parameter width) fit both the uniform and von Mises data best (YY, YN, NY).

	BIC				AIC			
Δ IC from VP-known	mean	s.e.m.	95% CI		mean	s.e.m.	95% CI	
FP-known	-31.58	6.41	-44.68	-21.63	-36.07	6.44	-36.11	-12.56
VP-inaccesible	-21.18	6.70	-44.26	20.39	-16.68	6.68	-48.84	-24.60
VP-discrete	-32.57	6.22	-36.62	10.81	-23.58	6.20	-32.55	-6.39

	BIC				AIC			
Δ IC from YY	mean	s.e.m.	95% CI		mean	s.e.m.	95% CI	
NY	-5.86	4.86	-17.57	1.50	-5.86	4.86	-17.80	1.35
YN	-10.23	5.27	-22.43	-2.17	-10.23	5.27	-22.5	-2.11
NN	-20.26	6.06	-33.92	-11.40	-25.40	6.04	-38.41	-15.55
TT	-39.72	6.25	-50.85	27.79	-44.87	6.26	-55.24	31.90

Table S1. Model comparison results using BIC and AIC.

B. Model comparison including von Mises and uniform distribution data.

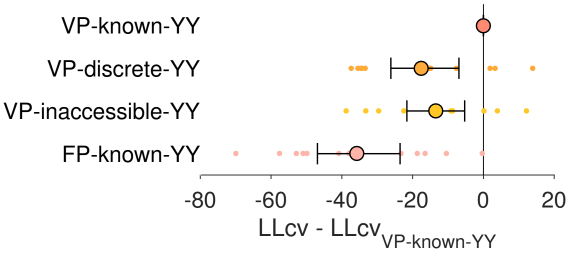

Fitted on all data				
Δ LLcv from VP-known-YY	mean	s.e.m.	95% CI	
VP-discrete-YY	-17.56	5.11	-26.15	-6.92
VP-inaccessible-YY	-13.46	4.42	-21.62	-5.30
FP-known-YY	-35.78	6.23	-46.90	-23.55

Fig. S10. Using the best fitting model of prior use (YY), we can redo the model comparison of main Figure 4 (uniform data only), by fitting the FP-known-YY, VP-inaccessible-YY, and VP-discrete-YY, models jointly on uniform and von Mises stimulus distribution trials. As in main Figure 4, the VP-known model fits best.

C. Our approximation to $p(\text{hit}|A, \hat{s}, x)$ is good.

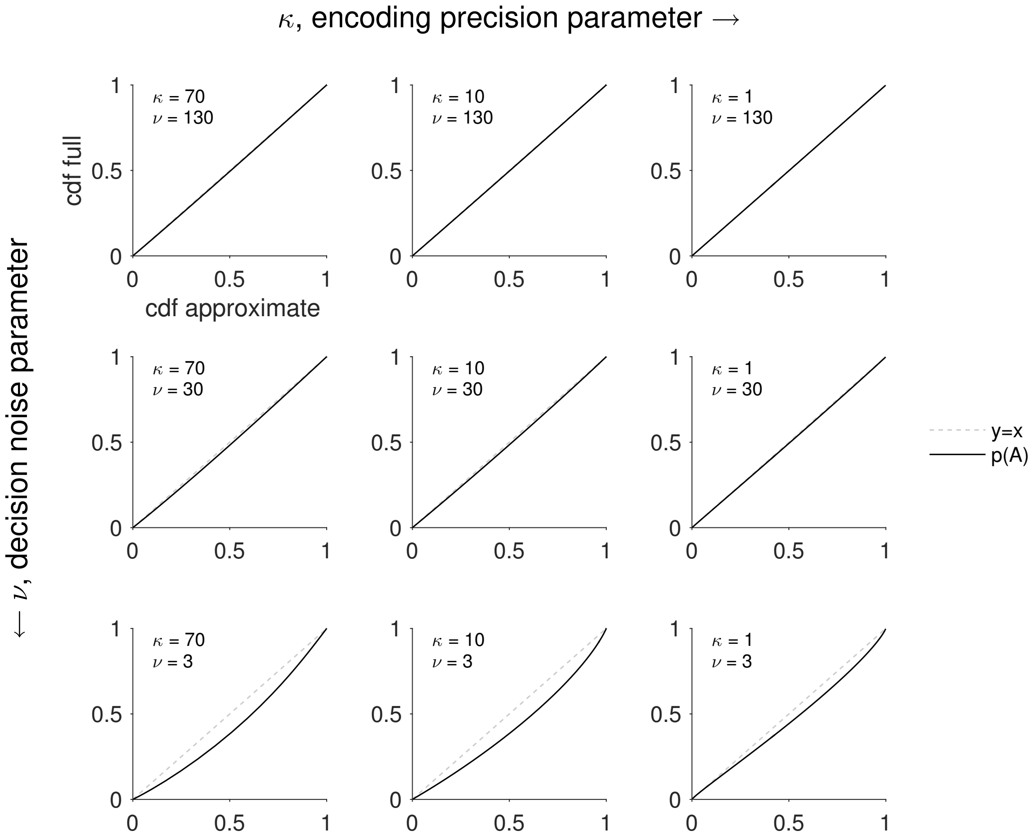

Fig. S11. To make our models more computationally tractable, we approximated the probability of obtaining reward in the model, the integral $p(\text{hit}|\hat{s}, x, \kappa)$, by assuming that $\hat{s}$ is equal to the mean of the posterior (see Methods). To check how much this approximation affects the model we simulated the distribution of arc sizes from the unapproximated (full) model, $p(A|\hat{s}, x, \kappa)$, and compare to our approximation which removes x and $\hat{s}$, $p(A|\kappa)$. This is done for for several sets of encoding precision parameters (left-right subplots) and decision noise parameters (up-down subplots). In this simulation we set the other model parameters at the mean of the participant data, $\alpha = 2$ and $\beta = 21$. We can visualize the difference between the predicted probability distributions by plotting the cumulative distribution function (cdf) of the approximation against the cdf of the full model (solid line, plotted with y=x the dotted line). The cdfs are generally similar, showing our approximation to $p(\text{hit}|A, \hat{s}, x, \kappa)$ with $p(\text{hit}|A, \kappa)$ is good. However, as decision noise increases, the cdf of the full model is greater than the cdf of the approximate, suggesting that arc sizes are sligHly underestimated under high decision noise.

D. Model parameters are all necessary.

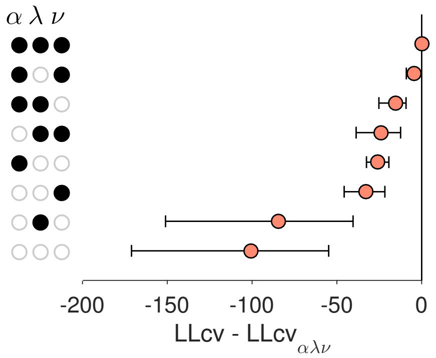

Fitted on uniform data					
Δ LLcv from full model	(α, λ, ν)	mean	s.e.m.	95% CI	
all knocked out		-100.57	29.94	-161.52	-32.74
α knocked in	(α)	-25.79	3.53	-32.74	-19.37
λ knocked in	(λ)	-84.41	28.54	-152.25	-41.00
ν knocked in	(ν)	-33.00	6.36	-45.82	-21.84
α knocked out	(λ, ν)	-23.87	6.81	-38.51	-12.61
λ knocked out	(α, ν)	-4.60	1.41	-9.24	-2.87
ν knocked out	(α, λ)	-15.44	4.13	-25.72	-9.26

Fig. S12. To test whether model parameters were necessary we compared the VP-known model with each combination of parameters ($\alpha\lambda\nu$) removed to the full model with all parameters. Comparisons were made using 10-fold cross validated log likelihood fitted on the uniform distribution data. Negative numbers represent worse fitting models. Presence (●) or absence (○) of model parameters is indicated in the y axis. Removing any model parameter (α, risk parameter, λ, lapse rate, ν stimulus estimate decision noise), worsens the fit of the model. In general, the more parameters removed, the worse a model is.

E. Parameters are consistent for individuals.

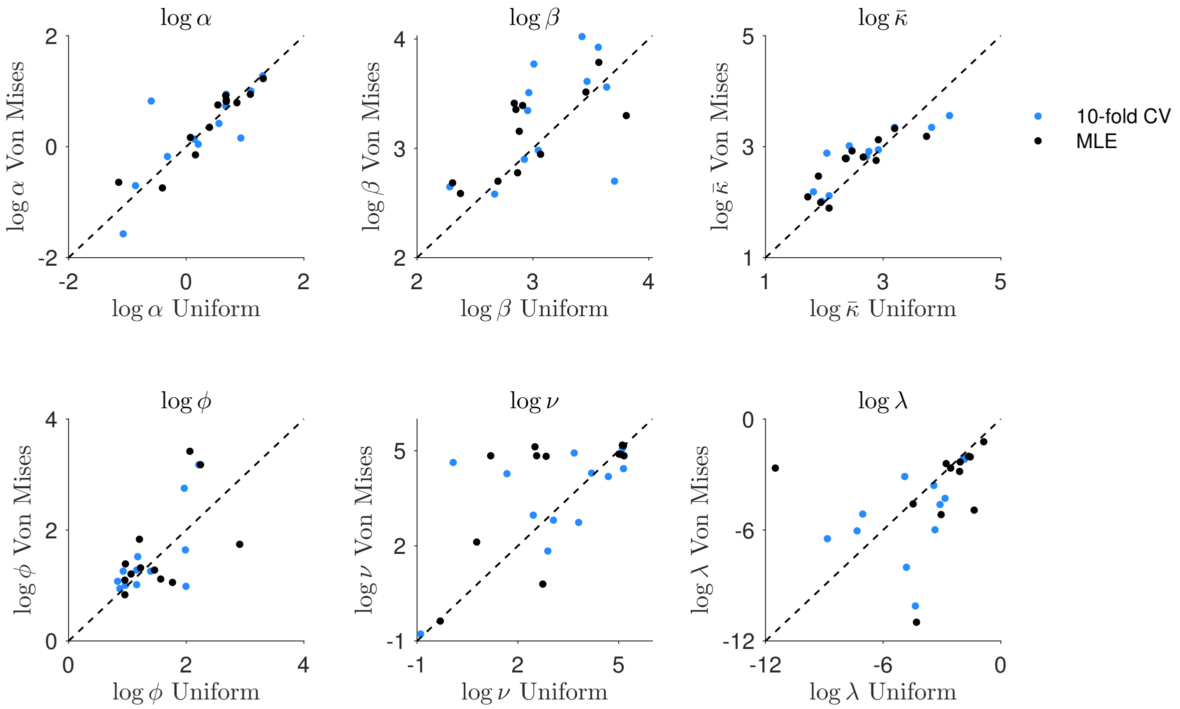

Fig. S13. To check if parameter estimates are consistent across stimulus distribution, we fit the best fitting models on either the uniform (VP-known) or von Mises data (YY, which is VP-known with an added prior), and compare the parameters. Plotted are the parameter estimates for uniform and von Mises data for all shared parameters ($[\alpha, \beta, \bar{\kappa}, \phi, \nu, \lambda]$). Both MLE (dark blue) or mean across folds of 10-fold cross validated parameters (ligH blue) predict similar parameters for each participant despite being fitted on different data (see below table). This suggests that changing the stimulus distribution does not change model parameters and that model parameters are capturing features of individual participant behavior. Plotted in log space for visualization due to the large ranges covered by fit parameters.

F. Delayed estimation model fits (no arc response).

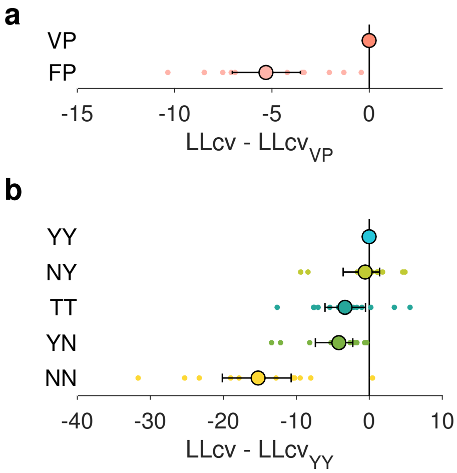

Fitted on stimulus estimate only

Δ LLcv from VP	mean	s.e.m.	95% CI	
FP	-5.30	0.093	-7.04	-3.54

Δ LLcv from YY	mean	s.e.m.	95% CI	
NY	-0.58	1.27	-3.58	1.42
TT	-3.34	1.48	-6.06	-0.52
YN	-4.20	1.32	-7.39	-2.26
NN	-15.20	2.52	-20.13	-10.70

BMS: TT	YY	YN	NY	NN
frequency: 0.21	0.24	0.08	0.41	0.07
pxp: 0.14	0.17	0.08	0.52	0.08

Fig. S14. Model comparison, fit on stimulus estimates but not arc responses. Without the arc report the data and task are analogous to a delayed estimation task. **(a)** Comparison of VP and FP models, fitted on uniform data (analogous to main Figure 4). VP model performs better than FP. VP-inaccessible and VP-discrete models become the same as VP-known model without an arc response and are thus not fit. **(b)** Comparison of prior use models, fitted on all data (analogous to main Figure 6). YY/NY models perform the best and the TT/YN model perform well. Performing Baysian Model Selection (BMS) yields model frequencies and protected exceedance probabilities (pxp) similar to those from models fitted on both estimates and arc responses. Participants are more likely to be using different models than all the same (random odds ratio = $3.57 \cdot 10^4$). Thus even without fitting the arc response our data supports that people have variable precision and can use prior information.

		model				
		YY	NY	NN	YN	TT
data	YY	0.00	-12.70	-35.60	-13.75	-30.30
	NY	17.26	0.00	-35.27	-29.10	-59.60
	NN	-5.59	-5.86	0.00	-6.19	-71.87
	YN	-2.91	-24.80	-24.80	0.00	-45.02
	TT	-6.30	-43.18	-81.50	-20.05	0.00

		model				
		YY	NY	NN	YN	TT
data	YY	0.00	-12.70	-40.66	-13.75	-35.37
	NY	-17.26	0.00	-40.33	-29.10	-64.67
	NN	-0.53	-0.79	0.00	-1.13	-71.87
	YN	-2.91	-24.80	-27.85	0.00	-50.08
	TT	-1.23	-38.1	-81.53	-14.98	0.00

G. Model distinguishability: recovery simulations.

To examine the confusability of models, we generated a confusion matrix by generating data from each of the models (YY,NY,NN,YN,TT) and fitting each model on the simulated data. We simulate 12 datasets based on the model parameters fitted to each of our 12 subjects and fit each dataset with each model. Plotted are the mean loglikelihood differences with a two sided t-test of the 12 datasets between the fitted model (x-axis) and the generating model (y-axis). Fitting a model on the data it generated always yields the maximum likelihood (the difference is 0.00). Furthermore the generating model is always substantially more likely (negative log likelihood difference) than any other model, except in the case of NN-YY and NN-NY when using AIC.

This is most likely due to the fact that NN is a model which is nested within the YY, YN and NY models. When the generating model is NN, then the YY, YN, and NY models all generate the same prediction (that the agent will not use the prior). Thus the likelihoods should only differ by the AIC difference (-1). The fact that NN-YY and NN-NY have differences of -0.52 and -0.79 respectively suggests that in the case of data generated with a flat prior distribution, our models may not perfectly recover when using AIC, perhaps due to imperfect optimization in fitting. However given the behavior and modeling evidence which demonstrates that participants use prior information, this is most likely irrelevant to the conclusions drawn in the text.

H. Model distinguishability: the KL divergence.

To examine model distinguishability we calculate the Kullback-Leibler (KL) divergence between models (10). This is the amount of information lost if you encode one probability distribution P with another Q.

$$\mathrm{D_{KL}}(P||Q) = \sum_i P_i \log(P_i) - P_i \log(Q_i)$$

When this is zero then $P = Q$. Note that this is non-symmetric, $\mathrm{D_{KL}}(P||Q) \neq \mathrm{D_{KL}}(Q||P)$. Since this is computationally difficult when P and Q are continuous, we use the unbiased approximation:

$$\mathrm{D_{KL}}(P||Q) = \log p(\mathrm{data}_P|P) - \log p(\mathrm{data}_P|Q)$$

where data_P is data sampled from P. To reduce variability we calculate $\mathrm{D_{KL}}(M_P||M_Q)$ as the mean of this approximation across 100 simulated datasets.

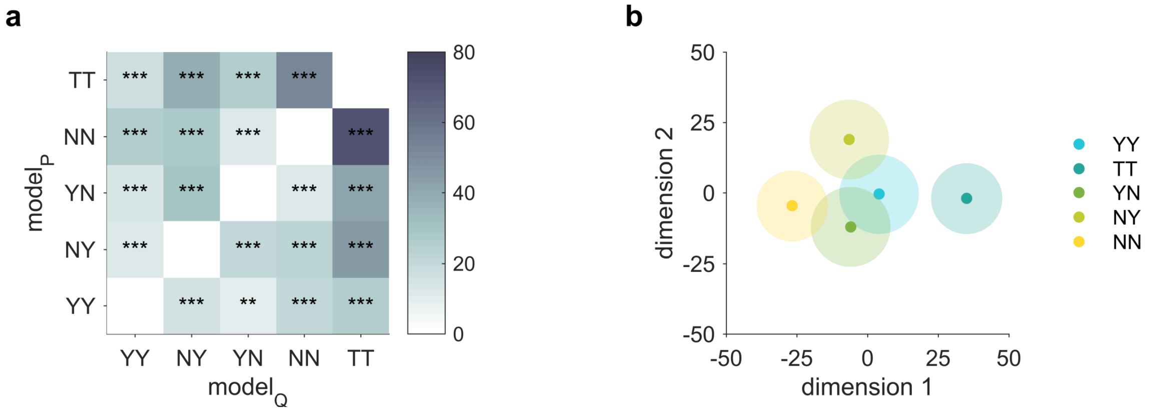

Fig. S15. **(a)** We calculate $\mathrm{D_{KL}}(M_P||M_Q)$ between the five prior-use models for each participant. The mean $\mathrm{D_{KL}}$ across participants is greater than zero for all models, showing that models generate distinct predictions. Furthermore the observed summed KLs across participants are unlikely given the expected distribution (see below, *,**,*** indicate $p < .05, .1, .001$ respectively). **(b)** We further visualize the differences between models by doing 2-dimensional multidimensional scaling (MDS), a method of visualizing the similarity between items in a dataset (similar to PCA). We apply 2-dimensional MDS using the symmetrized KL, $\mathrm{sD_{KL}} = \frac{1}{2}\mathrm{D_{KL}}(M_P||M_Q) + \frac{1}{2}\mathrm{D_{KL}}(M_Q||M_P)$, as the similarity matrix. Plotting this in a 2D plane, we see that the NN and TT models, and the NY and YN models are far apart suggesting that they are dissimilar. Interestingly the best fitting model, YY is similar to all other models. Filled area represents the area where the KL between the model and itself should fall for 95% of data simulated from that model (see below).

To assess the magnitude of $\mathrm{D_{KL}}$, we calculate the expected distribution of the KL between a model and itself, given that differences between the model and itself are caused by noisy parameter estimation. Let's consider a probability distribution

P, a vector of parameters θ with length n_θ, and the maximum likelihood estimate of parameters θ_{MLE}. If we assume that θ is Gaussian distributed with variance equal to the inverse fisher information (i.e. that the posterior over parameters is Gaussian), we can calculate the distribution of $\mathrm{D_{KL}}(P(\theta_{\mathrm{MLE}}), P(\theta))$. This follows a χ^2 distribution with n_θ degrees of freedom (11). Following this, across participants, the distribution of the sum of the KL is the sum of n_s χ^2 distributions with $df{=}n_\theta$, where n_s is the number of participants. This is a χ^2 distribution with $df{=}n_s n_\theta$. ($n_s{=}12, n_\theta{=}6$ or 7). Thus, the probability of the summed KL across participants is the inverse χ^2 cdf with $n_s n_\theta$, evaluated at $\sum \mathrm{D_{KL}}$. Furthermore, for a model with a parameter space of dimensionality n_θ, we can obtain the range in which the mean KL would fall for 95% of θ: the inverse χ^2 cdf of 0.95 with $df{=}n_\theta$.

I. The expected distribution of the KL divergence.

Since models have noisy parameter estimates, we would expect $\mathrm{D_{KL}}$ to be larger than zero. Let's assume that model$_Q$ is the same as model$_P$, but with noisy parameter estimates θ of the true parameters θ_{MLE}. Thus what we are actually calculating is $\mathrm{D_{KL}}(P \text{ with } \theta_{\mathrm{MLE}} || P \text{ with } \theta)$. If we assume that θ is Gaussian distributed around θ_{MLE} (i.e. that the posterior over parameters is Gaussian) and that the variance of this Gaussian is equal to the inverse fisher information (I_f), we can calculate the expected distribution of the KL divergence between the models with parameters θ_{MLE} and the noisy estimate θ: $\mathrm{D_{KL}}(p(D|\theta_{\mathrm{MLE}}), p(D|\theta))$.

First, a shorthand for the KL divergence

$$\mathrm{KL}(\theta_{\mathrm{MLE}}, \theta) = \mathrm{D_{KL}}(p(D|\theta_{\mathrm{MLE}}), p(D|\theta))$$

We will also need the definition of Fisher Information in terms of the KL divergence (it is the second derivative).

$$I_f = \nabla_\theta^2 \mathrm{KL}(\theta_{\mathrm{MLE}}, \theta)|_{\theta=\theta_{\mathrm{MLE}}} \qquad \text{the second derivative of KL}$$

We assume that the posterior over parameters is in the limit a Gaussian, with variance equal to the inverse fisher information (I_f)

$$p(\theta|D) \sim \mathcal{N}(\theta_{\mathrm{MLE}}, {I_f}^{-1}) \qquad \text{as } D_n \longrightarrow \infty$$

From the definition of KL above, we get the following relationship between $\mathrm{KL}(\theta_{\mathrm{MLE}}, \theta)$ and the Fisher information (Taylor expansion). The terms of this do not grow at a rate greater than $(\theta - \theta_{\mathrm{MLE}})^3$.

$$\mathrm{KL}(\theta_{\mathrm{MLE}}, \theta) = \left[\vec{\theta} - \vec{\theta}_{\mathrm{MLE}}\right]^T I_f \left[\vec{\theta} - \vec{\theta}_{\mathrm{MLE}}\right] + \mathcal{O}((\theta - \theta_{\mathrm{MLE}})^3)$$

As the number of samples of θ grows to infinity, the Fisher Information I_f increases linearly, while θ is normally distributed with mean θ_{MLE} and variance I_f^{-1} (local asymptotic normality, since under the model all trials are independent and identically distributed). In this limit, the second term in this equation decays to zero whereas the first term converges in distribution to a χ^2 with n_θ degrees of freedom:

$$\begin{aligned}
\left[\vec{\theta} - \vec{\theta}_{\mathrm{MLE}}\right]^T I_f \left[\vec{\theta} - \vec{\theta}_{\mathrm{MLE}}\right] &= \vec{x}^T I_f \vec{x} && \text{with } \vec{x} = (\vec{\theta} - \vec{\theta}_{\mathrm{MLE}}) \sim \mathcal{N}(0, I_f^{-1}) \\
&= \vec{y}^T \vec{y} && \text{with } \vec{y} = \sqrt{I_f}\vec{x} \sim \mathcal{N}(0, \mathrm{I}) \\
&= \sum_{i=1}^{n_\theta} y_i^2 && \text{with } y_i \sim \mathcal{N}(0, 1) \text{ for all } i
\end{aligned}$$

A sum of n_θ independent standard normally distributed variables follows a χ^2 distribution with n_θ degrees of freedom.

$$\mathrm{KL}(\theta_{\mathrm{MLE}}, \theta) \sim \chi^2(n_\theta)$$

Specifically, the expected value of $\mathrm{KL}(\theta_{\mathrm{MLE}}, \theta)$ is n_θ and its variance is $2n_\theta$. This allows us to estimate the probability of an observed KL divergence, $\mathrm{D_{KL}}(P||Q)$, under the null hypothesis that Q is noisy estimate of P, $p(\mathrm{D_{KL}}) = \chi^2(x = \mathrm{D_{KL}}, df = n_\theta)$. We can also use this expected distribution of $\mathrm{KL}(\theta_{\mathrm{MLE}}, \theta)$ to calculate the range in which $\mathrm{KL}(\theta_{\mathrm{MLE}}, \theta)$ would fall for 95% of the data generated from P with number of parameters n_θ, this is the inverse χ^2 cdf of 0.95 with degrees of freedom equal to n_θ. Across participants, the distribution of the $\sum \mathrm{KL}(\theta_{\mathrm{MLE}}, \theta)$ is the sum of n_s χ^2 distributions, a χ^2 distribution with degrees of freedom equal to $n_s \cdot n_\theta$. Thus the probability of the observed summed $\mathrm{D_{KL}}(P, Q)$ across participants then is 1-χ^2cdf$(n_s n_\theta)$ evaluated at $\sum \mathrm{D_{KL}}(P, Q)$.

4. Misc.

A. Table of best model parameters.

Uniform data (VP-known model)						Uniform and von Mises data (YY model)						
α	β	$\bar{\kappa}$	ϕ	ν	λ	α	β	$\bar{\kappa}$	ϕ	ν	λ	κ_w
1.49	17.6	18.5	2.61	17.2	0.196	1.45	16.9	21.7	2.73	16.2	0.170	0.0699
1.98	21.4	10.6	3.33	15.6	0.127	2.23	19.2	16.3	3.12	5.17	0.0829	1.03
1.08	10.1	11.8	5.86	2.19	0.415	1.08	11.5	15.3	4.02	2.26	0.370	0.317
0.666	17.9	42.2	2.63	0.737	0.0621	0.59	19.6	30.7	3.20	0.735	0.0851	$3 \cdot 10^{-10}$
2.99	35.4	6.91	7.83	167	0.0113	2.82	38.3	6.94	12.9	171	0.0149	0.663
1.71	18.3	14.4	2.61	151	0.0475	1.94	23.7	15.5	2.46	137	0.0586	0.553
2.37	17.1	6.70	18.3	173	0.0785	2.45	24.9	9.61	5.62	19.1	0.120	0.133
1.98	14.8	24.4	3.40	3.28	0.122	2.02	14.5	28.0	3.51	3.97	0.0945	0.205
1.95	31.8	8.00	9.46	171	0.0137	2.19	31.3	7.52	12.4	172	0.00821	0.417
1.17	10.8	10.7	4.32	12.9	0.212	1.04	11.7	13.6	3.66	31.8	0.173	0.0693
0.317	17.3	5.57	4.79	162	$1 \cdot 10^{-5}$	0.363	19.1	6.48	3.99	159	0.0114	0.574
3.71	44.9	17.8	2.90	12.3	0.261	3.46	28.9	16.0	3.12	165	0.0707	0.305

Table S3. Tables of MLE parameter estimates of the best fitting models for both uniform stimulus distribution data (VP-known model) and for von Mises and uniform stimulus distribution data (YY model) for all 12 participants.

B. Model fits.

Model fits obtained by simulating data using fitted parameters on uniform stimulus distribution trials (top) and both uniform and von Mises stimulus distribution trials (bottom). Black error bars represent data and colored fill represents model fits, plotting conventions are the same as in main Figures 2 and 5. To get representative predictions, plotted predictions were averaged across 20 simulations for each participant.

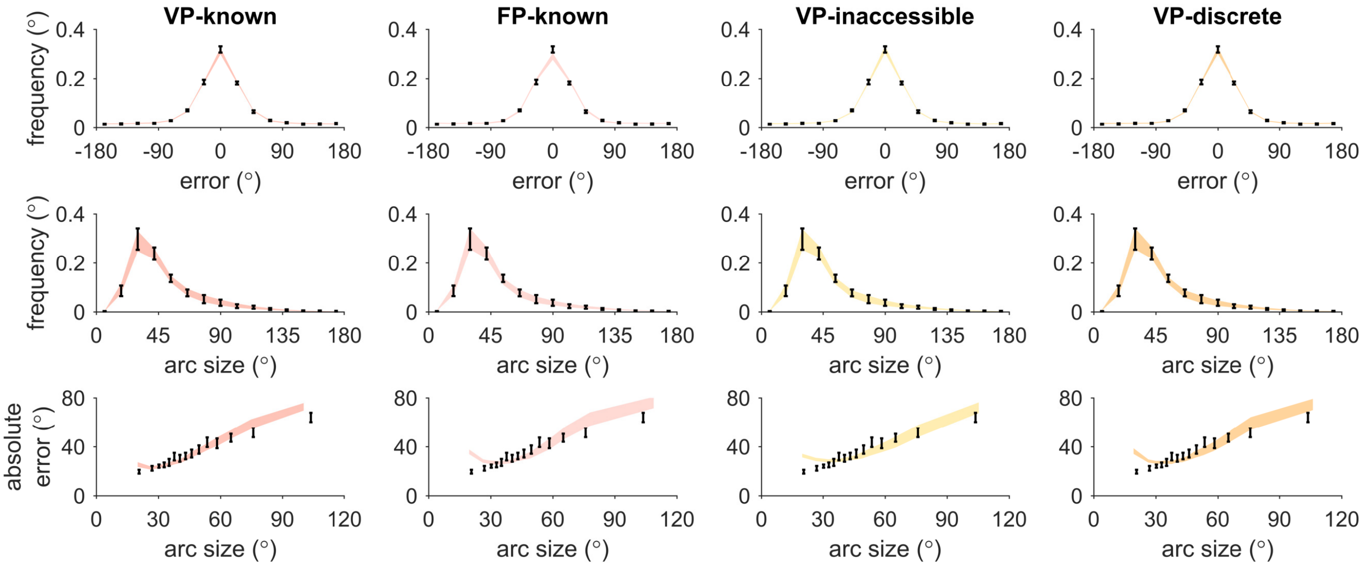

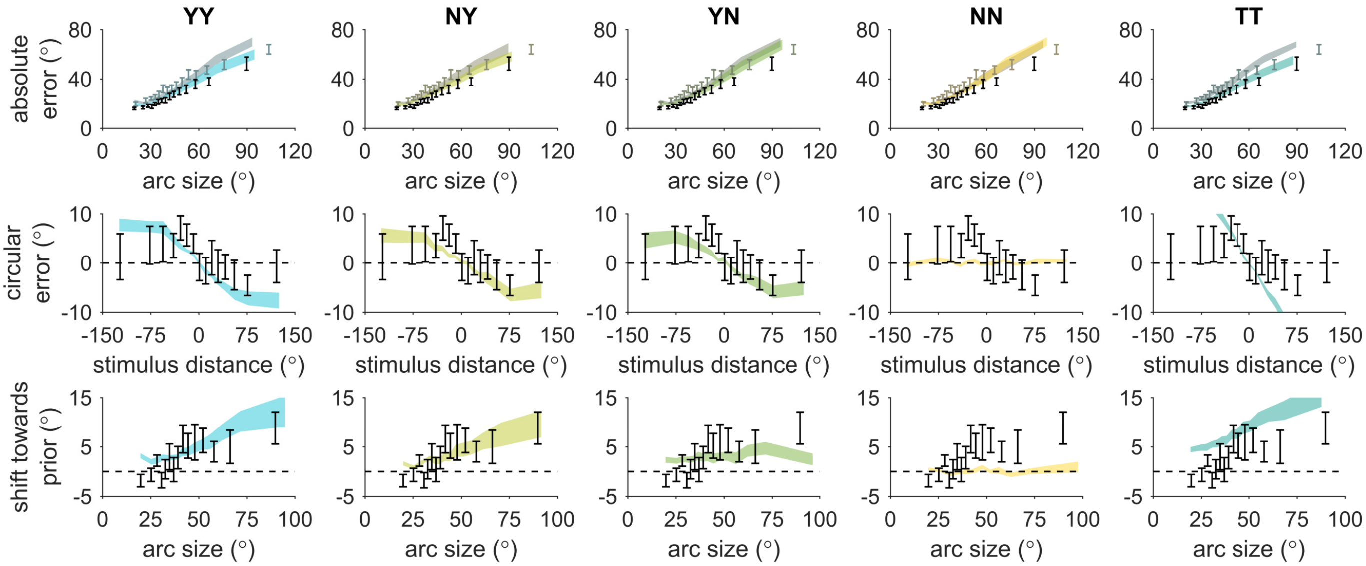

References

1. van den Berg R, Shin H, Chou WC, George R, Ma WJ (2012) Variability in encoding precision accounts for visual short-term memory limitations. *Proceedings of the National Academy of Sciences* 109(22):8780–8785.
2. van den Berg R, Awh E, Ma WJ (2014) Factorial comparison of working memory models. *Psychological Review* 121(1):124–149.
3. Fougnie D, Suchow JW, Alvarez GA (2012) Variability in the quality of visual working memory. *Nature Communications* 3:1229.
4. Murray RF, Morgenstern Y (2010) Cue combination on the circle and the sphere. *Journal of Vision* 10(11):15–15.
5. Acerbi L, Vijayakumar S, Wolpert DM (2014) On the origins of suboptimality in human probabilistic inference. *PLoS Computational Biology* 10(6):e1003661.
6. Kahneman D, Tversky A (1979) Prospect theory: An analysis of decision under risk. *Econometrica* 47(2):263.
7. Zhang W, Luck SJ (2008) Discrete fixed-resolution representations in visual working memory. *Nature* 453(7192):233–235.

8. Stephan KE, Penny WD, Daunizeau J, Moran RJ, Friston KJ (2009) Bayesian model selection for group studies. *NeuroImage* 46(4):1004–1017.
9. Rigoux L, Stephan KE, Friston KJ, Daunizeau J (2014) Bayesian model selection for group studies – revisited. *NeuroImage* 84:971–985.
10. Thomas M. Cover JAT (2006) *Elements of Information Theory.* (Wiley).
11. Salicrú M, Morales D, Menéndez ML, Pardo L (1994) On the applications of divergence type measures in testing statistical hypotheses. *Journal of Multivariate Analysis* 51:372–391.
